# Evolution of Hybrid Hydrogels: Next-Generation Biomaterials for Drug Delivery and Tissue Engineering

**DOI:** 10.3390/gels10040216

**Published:** 2024-03-22

**Authors:** Md Mohosin Rana, Hector De la Hoz Siegler

**Affiliations:** 1 Department of Pathology and Laboratory Medicine, Faculty of Medicine, University of British Columbia, Vancouver, BC V6T 1Z7, Canada; mohosin.rana@ubc.ca; 2 Centre for Blood Research, Faculty of Medicine, University of British Columbia, Vancouver, BC V6T 1Z3, Canada; 3 Department of Chemical and Petroleum Engineering, Schulich School of Engineering, University of Calgary, Calgary, AB T2N 1N4, Canada

**Keywords:** hybrid hydrogels, biomaterials, synthetic polymers, drug delivery, tissue engineering

## Abstract

Hydrogels, being hydrophilic polymer networks capable of absorbing and retaining aqueous fluids, hold significant promise in biomedical applications owing to their high water content, permeability, and structural similarity to the extracellular matrix. Recent chemical advancements have bolstered their versatility, facilitating the integration of the molecules guiding cellular activities and enabling their controlled activation under time constraints. However, conventional synthetic hydrogels suffer from inherent weaknesses such as heterogeneity and network imperfections, which adversely affect their mechanical properties, diffusion rates, and biological activity. In response to these challenges, hybrid hydrogels have emerged, aiming to enhance their strength, drug release efficiency, and therapeutic effectiveness. These hybrid hydrogels, featuring improved formulations, are tailored for controlled drug release and tissue regeneration across both soft and hard tissues. The scientific community has increasingly recognized the versatile characteristics of hybrid hydrogels, particularly in the biomedical sector. This comprehensive review delves into recent advancements in hybrid hydrogel systems, covering the diverse types, modification strategies, and the integration of nano/microstructures. The discussion includes innovative fabrication techniques such as click reactions, 3D printing, and photopatterning alongside the elucidation of the release mechanisms of bioactive molecules. By addressing challenges, the review underscores diverse biomedical applications and envisages a promising future for hybrid hydrogels across various domains in the biomedical field.

## 1. Introduction

Hydrogels are hydrophilic polymer networks capable of absorbing, swelling, and retaining substantial amounts of aqueous fluids [1]. Their suitability for biological applications stems from their high water content, permeability, tunable viscoelasticity, and structural resemblance to the extracellular matrix. In 1960, Wichterle and Lim highlighted the potential of hydrogels for biomedical applications, establishing their significance in tissue engineering and drug delivery [2]. The progress in hydrogel chemistry has enhanced the versatility and functionality of hydrogels, allowing for the integration of molecules that guide cellular activities and enabling the time-controlled activation of the hydrogel system and the incorporation of transporters, such as nanoparticles and nano/microstructure domains, into the hydrogel network [3].

Although hydrogels possess inherent properties that are useful for medical purposes, improvements are needed in their strength, release kinetics, stability, and degradability [2]. Conventional synthetic hydrogels often exhibit a significant heterogeneity and network imperfections, such as dangling chain ends, entanglements, and phase-separated regions [4], impacting the mechanical properties, diffusion rates, and biological activity of the encapsulated molecules [5]. Consequently, hybrid hydrogels have been developed to address these challenges, aiming to improve the formulations and expand their applications, including controlled drug release and the regeneration of soft and hard tissues and organs [6].

The definition of hybrid hydrogels is subject to an ongoing debate, with three primary interpretations. One perspective defines them as complexes resulting from the intricate crosslinking of numerous nanogels, achieved through either chemical or physical means [7]. In a second interpretation, the term is used to characterize systems developed by combining diverse polymers and/or integrating nanoparticles, including plasmonic, magnetic, and carbonaceous nanoparticles, among others [3,8]. In another interpretation, hybrid hydrogels consist of building blocks that are chemically, functionally, and morphologically distinct, originating from at least two different classes of molecules [3,9]. These classes may include biologically active polymers like polysaccharides and/or proteins, peptides, or nano/microstructures, connected by physical or chemical linkages [6]. The hybridization process can manifest at varying levels, occurring either at the molecular level or on a microscopic scale, contingent upon the size and nature of the building blocks.

Due to their versatile characteristics, hybrid hydrogels have garnered increasing attention within the scientific community, particularly for their diverse applications in the biomedical sector. Recent advancements in hybrid hydrogels have broadened their potential uses, especially in the delivery of therapeutics and regenerative medicine. This review explores the recent progress in advanced hybrid hydrogel systems, encompassing the different types, modification strategies, and the integration of nano/microstructures into hybrid hydrogels. Furthermore, it discusses innovative techniques, including click reactions, 3D printing, and photopatterning for material fabrication, along with the release mechanisms of bioactive molecules to achieve desired outcomes. Lastly, by addressing challenges, the review emphasizes diverse biomedical applications and provides insights into the promising future of hybrid hydrogels in various biomedical fields.

## 2. Hydrogel: Basic Architecture

Hydrogels comprise a three-dimensional (3D) structure capable of absorbing and swelling in water, primarily due to the presence of hydrophilic groups like −NH_2_, –COOH, –OH, –CONH_2_, –CONH, and –SO_3_H [10]. The network is typically formed by crosslinked polymer chains, established through the crosslinking of colloidal clusters. Their flexibility and softness arise from their high-water absorption capacity [11,12]. Hydrogel design involves the chemical or physical crosslinking of natural or synthetic polymer chains, resulting in a high porosity, favorable mechanical properties, and hydrophilicity [1,11,13], making them well-suited for various applications including biosensing, tissue regeneration, separation and purification, and tissue engineering [14].

**Figure 1:**
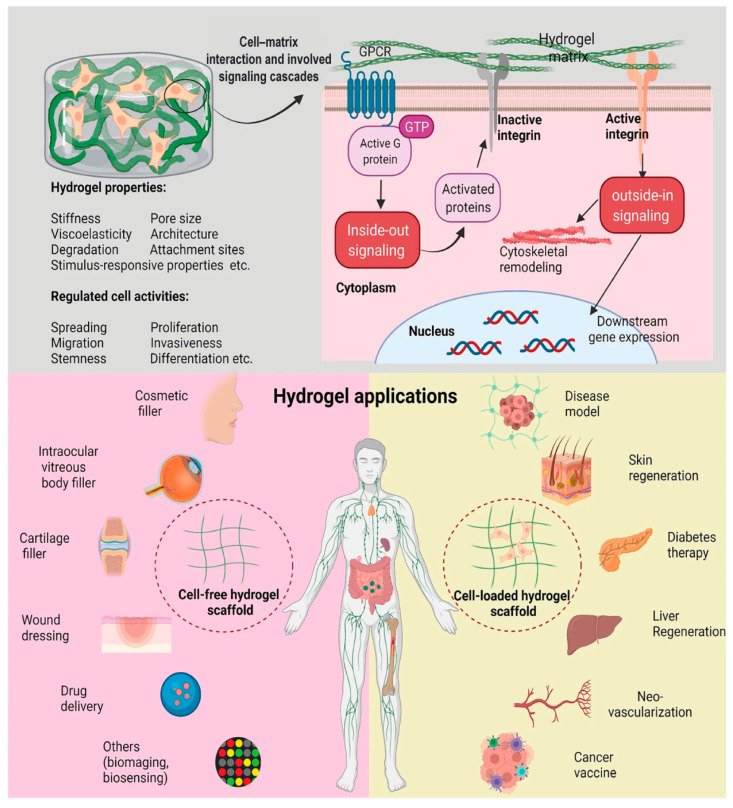
Schematic images depict the interactions between cells and hydrogel matrices, revealing how the physicochemical properties of hydrogels influence the cell biology by triggering corresponding signaling cascades, such as inside-out and outside-in signaling. Additionally, the images illustrate the diverse biomedical applications of both cell-free and cell-loaded hydrogels. Reproduced with permission from Cao et al. [23].

Hydrogels play a crucial role in tissue engineering applications, as they can provide physical support for cell growth and facilitate essential cell–matrix interactions by triggering signaling cascades (e.g., inside-out and outside-in signaling) (see Figure 1). Moreover, they create a biochemically suitable environment for cell growth and differentiation [15,16]. To meet the diverse requirements of biological environments, there is growing interest in multicomponent hybrid hydrogels [9], offering a range of properties that are necessary for optimizing the material’s performance in biological applications.

Various physical or chemical methods can be utilized to fabricate hybrid hydrogels. For instance, bioactive peptides and proteins can be combined with synthetic polymers through conjugation strategies such as click reactions or radical polymerization [17,18], offering a wide range of possibilities for creating hybrid hydrogels. Click reactions provide a straightforward pathway for crafting polymers and hydrogel networks with a high specificity, enabling precise structural control and pattern design [19]. Furthermore, these non-toxic and gentle reactions facilitate the in vivo formation of hydrogels and encapsulation of cells [20,21]. Additionally, the strategic utilization of peptide and protein networks induced by self-assembly can enhance the effectiveness of physically based methods for synthesizing self-assembled hydrogels [22].

Hydrogel scaffolds can be crafted using both natural and synthetic polymers. Biomimetic materials, especially structural proteins like elastin and collagen, exhibit mechanical properties akin to those found in native tissues [24]. Conversely, synthetic materials showcase diverse chemical properties and molecular structures [25]. The appeal of hybrid polymer scaffolds lies in their ability to mimic the extracellular matrix (ECM), generating significant interest [26]. Moreover, composite hybrid hydrogel networks are mechanically more robust due to additional mechanical reinforcements and the incorporation of a second phase (such as nano- and microparticles loaded with a drug) into the hydrogel matrix [27], enhancing the drug delivery performance.

The effectiveness of biochemically inert polymers in controlling the cell behavior might be limited due to the lack of interactions between the cells and hydrogel. Consequently, intentionally designing and manufacturing hybrid hydrogels to facilitate various biological functions is advisable [28]. In addition to enabling cell-mediated degradation and adhesion, hydrogels can support cell growth and differentiation by incorporating bio-functional biomolecules such as signaling molecules and growth factors [29]. Through the integration of controlled delivery systems for therapeutic agents and DNA, coupled with the regulated release of biomolecules that influence immune responses, hydrogels could extend their functionalities from tissue regeneration to encompass applications in gene therapy and cancer treatment.

## 3. Polymers Used in Hybrid Hydrogels

The development of hybrid hydrogels aims to address the challenges associated with the current formulations and expand their applications beyond drug delivery to tissue regeneration [2,3,6]. Hybrid hydrogels consist of diverse building blocks, such as biologically active proteins, peptides, or nano/microstructures, exhibiting distinct chemical, functional, and morphological characteristics. These elements are interconnected through physical or chemical methods. This section provides an overview of the natural and synthetic polymers commonly utilized in the fabrication of hybrid hydrogels, highlighting their characteristics and applications in biomedical research and technology.

### 3.1. Natural Polymers

Derived from natural sources, natural polymers ensure a biomimetic structure and bioactivity. Their key attributes include biocompatibility, a 3D geometry, non-toxic biodegradation, and an intrinsic structural likeness [30]. Scaffolds made from diverse biodegradable materials are widely employed in tissue engineering due to these advantageous properties. Natural polymers are broadly classified into polysaccharide and protein-based categories based on their monomeric units and structure.

#### 3.1.1. Polysaccharide-Based Polymers

Polysaccharides, consisting of various monosaccharide units linked by O-glycosidic bonds, successfully mimic the extracellular microenvironment [31] and can easily undergo biological degradation, minimizing the risk of bioaccumulation. Agarose, alginate, chitosan, cellulose, carrageenan, chondroitin sulfate, dextran, heparin, hyaluronic acid, pullulan, and starch have been researched as building blocks for hydrogels [32,33,34,35].

*Agarose* gels offer a high biocompatibility and non-immunogenicity [36], successfully mimicking the extracellular matrix (ECM) and tissue properties crucial for transporting cell growth factors to injured areas [37,38]. Blends of agarose and keratin are non-toxic [39], supporting viable cell growth in wound healing and skin regeneration studies. Moreover, the numerous pendant groups in agarose facilitate robust hydrogen bonding with drugs and bioactive molecules, enhancing its efficacy in drug delivery [40].

*Alginate*, an anionic polysaccharide derived from bacteria, brown algae, and kelp [41], possesses nonimmunogenic, biodegradable, and biocompatible characteristics. Its pH-dependent viscosity [42], mucoadhesive properties, and ability to differentiate chondrocytes make it valuable in crafting tissue engineering scaffolds [43,44,45]. Alginate–chitosan and alginate–chitosan–collagen formulations have been used for skin replacements and drug delivery systems [46,47,48]. Alginate–collagen scaffolds with curcumin nanoparticles have been found to promote wound healing [49].

*Cellulose* showcases exceptional mechanical strength owing to robust hydrogen bonding and cohesion [50], presenting a stable framework adaptable to various biocompatible forms [51]. Combining cellulose with zinc oxide nanoparticles boosts the mechanical strength and antibacterial properties, aiding in wound healing [52]. Carboxymethyl cellulose (CMC) serves as an effective scaffold material for regenerating bone, cartilage, and skin [53]. Electrospun cellulose extracted from Ulva lactuca, along with polylactide and polydioxanone, generates nanofibrous scaffolds, fostering cellular growth and angiogenesis [54].

*Chitosan*, derived from the chitin in arthropods, mollusks, and fungi, offers hydrophilicity, homeostasis, non-toxicity, mucoadhesion, and an ECM-like structure [55]. Coating biomaterials with chitosan enhances their biocompatibility [56]. The 3D scaffolds combining chitosan hydrochloride, collagen, CMC, and β-glycerophosphate enable the sustained release of rosuvastatin, promoting wound healing [57], which is further enhanced by mesenchymal stem cell incorporation in albino rats.

*Carrageenan* is a linear polysaccharide obtained from red seaweed that consists of α-1,3 and β-1,4-glycosidic bonded D-galactose and 3,6-anhydro-galactose. Carrageenan dissolves in water, yielding a thick solution whose viscosity is contingent on the concentration, temperature, and molecular weight [58]. It undergoes depolymerization through acid-catalyzed hydrolysis [59]. Carrageenan hydrogels have been extensively applied in drug delivery systems and tissue engineering [60,61].

*Chondroitin* sulfate, an anionic, unbranched polysaccharide found in joint cartilage, exhibits anti-inflammatory, biodegradable, nutrient-retentive, and cartilage-restorative properties [62].

*Dextran*, a high molecular-weight polysaccharide composed of α-1,6-linked glucose in the backbone and α-1,4-linked glucose in the side chains, exhibits varying structures depending on the source microbial strains [63]. It is biodegradable and biocompatible and is soluble in water, DMSO, and glycerol, but insoluble in alcohols and ketones [64]. Dextran hydrogel-based scaffolds demonstrated their effectiveness in dermal regeneration and promoting skin appendage growth in mice [65].

*Heparin* is a linear, negatively charged polysaccharide formed by β(1 → 4) bonded D-glucuronic, L-iduronic, D-N-acetyl glucosamine, and O- and N-sulfated glucosamine [66]. Its structural richness, with numerous carboxyl and sulfate groups, facilitates diverse biological interactions, yielding anti-coagulant, anticancer, anti-inflammatory, and anti-angiogenic effects [67].

*Hyaluronic acid* (HA), a negatively charged glycosaminoglycan, comprises N-acetyl-d-glucosamine and d-glucuronic acid units linked by β(1 → 4) and β(1 → 3) glycosidic bonds [68]. It is synthesized by hyaluronan synthases in cell membranes and is naturally found in synovial fluid and connective, epithelial, and neural tissues. As a crucial connective tissue component, hyaluronic acid aids cell growth and differentiation [69]. It is non-adhesive, hydrophilic, and biodegradable in both solution and hydrogel forms.

*Pullulan*, a polysaccharide consisting of maltotriose units connected by α-1,4 glycosidic bonds, is biocompatible, non-toxic, non-mutagenic, non-immunogenic, and biodegradable [70]. Pullulan-based hydrogels sustain the bioactivity in drug delivery, offering adjustable release profiles [70]. In tissue engineering, the focus lies on multifunctional pullulan-based 3D scaffolds for efficient tissue regeneration [71].

*Starch*, comprising α-amylose and amylopectin and being linked by glycosidic bonds, is cost-effective but lacks strong polymer interactions. Combining it with other polymers is essential due to subpar mechanical properties [72]. Light-curable, starch-based hydrogels that incorporate Fe_3_O_4_ nanoparticles offer controlled quercetin release, an improved bioavailability, and enhanced mechanical properties through crosslinking under blue light [73].

#### 3.1.2. Protein-Based Polymers

Under appropriate conditions, protein-based polymers, derived from natural α-L amino acids including collagen, elastin, fibrin, gelatin, and keratin, can exhibit self-organization and entanglement to create 3D hydrogels. These are the result of nanometer-wide and micrometer-long fibrils formed through intermolecular and/or intramolecular interactions (e.g., H bonds, electrostatic forces, and hydrophobic effects) [74].

*Collagen* exhibits a high compatibility with chitosan, hyaluronic acid, and chondroitin sulfate, allowing for their modification and resulting in enhanced biological and mechanical properties. Collagen offers a good biocompatibility, low antigenicity, and high mechanical strength, facilitating cell functions and ECM production [75]. It can be easily modified, and varying its concentration enables the customization of scaffold features.

*Elastin* is a hydrophobic ECM protein with diverse biomaterial applications, and includes soluble and insoluble forms in conjunction with other polymers [30,76]. Collagen–elastin scaffolds offer dermal substitutes for skin renewal [77]. Tri-polymer scaffolds of elastin, collagen, and polycaprolactone (PCL) nanofibers show promise in severe burn care [78]. Silk–elastin-like protein-based polymers (SELPs) create injectable hydrogels with thermal-responsive properties, ideal for various biomedical deliveries, such as through a cell, DNA, protein, and therapeutic agents [79].

*Fibrin*, a resilient protein formed from fibrinogen in blood plasma, can serve as a tissue scaffold due to its high biocompatibility and customizable binding sites [80]. Unfortunately, fibrin lacks the mechanical strength necessary for standalone tissue regeneration. Thus, fibrin scaffolds need to be combined with synthetic materials, such as polyglycolic acid (PGA), polylactic acid (PLA), PCL, and polyvinyl alcohol (PVA), or other natural polymers, such as hyaluronic acid, alginate, or collagen [81].

*Gelatin*, derived from collagen hydrolysis, is valuable for robust biomedical hydrogels, boasting ample functional groups and easy crosslinking [82]. Water-soluble, non-immunogenic, and amphoteric at 37 °C, it is ideal for crafting contact lenses, tissue engineering matrices, and drug delivery [82,83]. Its enzymatic breakdown enables a gradual biological agent release, and its charge can be adjusted for a controlled delivery. Methacrylamide-modified gelatin (GelMA) offers customizable mechanical properties for recreating stem cell niches in tissue engineering [84].

*Keratin* is a naturally abundant structural protein that exhibits self-assembly properties [85]. Keratin-based hydrogels with a high cysteine content support cell proliferation, forming porous gels that are suitable for prosthetic injection and skin/tissue regeneration [86]. These hydrogels are stable and mechanically robust even without crosslinking.

### 3.2. Synthetic Polymers

**Figure 2:**
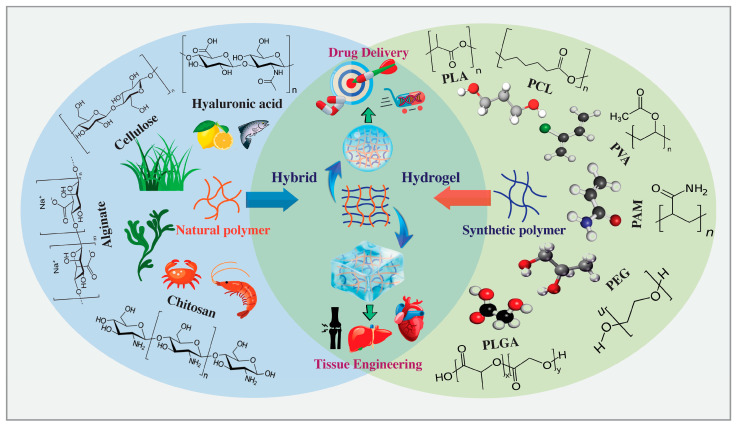
Hybrid hydrogels, composed of a combination of natural and synthetic polymers, serve a dual purpose in drug delivery systems and as scaffolds for tissue engineering applications.

Synthetic polymers such as poly(ethylene glycol) (PEG), poly(acrylamide) (PAM), PVA, PLA, and PCL offer diverse structures and controlled degradation, making them advantageous for various biomedical applications. Synthetic polymers offer a customizable mechanical stability but lack cell adhesion sites, requiring chemical modifications for enhanced cellular interactions [87]. A strategic approach involves combining synthetic polymers with natural counterparts to create hybrid hydrogels with diverse properties, inspired by nature and suitable for versatile applications (Figure 2).

*Poly(ethylene glycol)* (PEG) features hydrophilic -OH groups in its non-degradable, biocompatible chain. PEG excels in bone tissue engineering due to its biocompatibility [88]. For instance, a 3D scaffold combining PEG-methyl ether methacrylate, calcium-alginate, and *M. olifera* exhibited structural stability, anti-inflammatory traits, and antioxidant abilities [89]. PEG also shields drug delivery systems from an immune response, while enhancing their physicochemical properties.

*Poly(acrylamide) (PAM)*, a hydrophilic and non-toxic synthetic polymer, is extensively employed in hydrogel synthesis for tissue engineering, drug delivery, and biosensors [16,90]. However, its application is constrained by the release of acidic byproducts during degradation in physiological fluids, which impacts cell viability [91].

*Poly-β-hydroxybutyrate (PHB)*, a biodegradable homopolymer derived from microorganisms, has been used in cardiovascular surgery for tissue repair [92] and spinal cord injury research [93]. Electrospun nanofibers composed of PHB and PVA demonstrate versatility in skin tissue engineering, highlighting their potential in regenerative medicine [94].

*Polyvinyl alcohol (PVA)*, derived from poly(vinyl acetate) hydrolysis, has favorable attributes including biodegradability, biocompatibility, and non-toxicity. PVA’s hydrophilicity and solubility are determined by its hydrolysis degree and molecular weight [95]. PVA has been combined with chitosan and PHB in nanofiber production for wound healing and tissue engineering [94,96]. Its low protein adsorption and biocompatibility make it suitable for tissue adhesive applications.

*Polylactic-co-glycolic acid (PLGA)*, a biocompatible and biodegradable copolymer [97], has been used in the preparation of microspheres for drug delivery [98]. It efficiently controls the pH and ensures a sustained release of Mg^2+^ ions and oxygen [99]. The current focus is on PLGA-based thermo-sensitive hydrogels for precise drug delivery control. However, their use in tissue regeneration is limited by their mechanical weakness, often mitigated by blending with other polymers.

*Polycaprolactone (PCL)*, an aliphatic, biocompatible, and biodegradable polyester is commonly used alone or in combination with other polymers for gelation, resulting in hydrogels with specific characteristics. While effective in multiphasic tissue engineering for mesenchymal stem cell transplantation, PCL’s drawbacks include its low bioactivity and high hydrophobicity, hindering cell affinity and tissue regeneration [76].

*Polylactic acid (PLA)* is a semi-crystalline, hydrophobic, aliphatic, and biodegradable material. Despite its biocompatibility and degradability, its long-term use is controversial due to lactic acid release during hydrolytic degradation, lowering the local pH and leading to inflammation and cell death [100]. Blending PLA with PCL enhances the degradability and mechanical properties [101]. In bone regeneration studies, PCL exhibits stability for 6 months and complete degradation in 3–4 years without harmful byproducts [102,103].

*Resilins* are elastomeric proteins present in the exoskeletons of many arthropods that form di- and trityrosine crosslinked hydrogels and boast exceptional mechanical traits including a low stiffness, high resilience, and efficient energy storage [104]. Engineered resilin-like polypeptides (RLPs), which replicate native resilin’s advantages, are promising to design biomaterials in tissue engineering. Recombinant RLPs such as RLP–PEG closely mimic natural resilin’s mechanical prowess, hinting at their utility in demanding tissue engineering endeavors [104].

*Poly(glycerol sebacate)* (PGS), a glycerol–ester polymer derived from glycerol and sebacic acid, boasts FDA-approved constituents [105]. Initially developed for its biodegradability and enhanced mechanical properties, PGS offers excellent biocompatibility, which results in low swelling, surface degradation, and mild inflammatory reactions. Its versatility spans from thermoset elastomer to customizable resin forms, adaptable for various applications including coatings and luminal structures [105,106].

The combination of synthetic polymers with natural counterparts results in a diverse set of characteristics, where even minor alterations in the chemical compositions or fabrication processes can result in the creation of entirely new biomaterials.

### 3.3. Modification of Polymers in Hybrid Hydrogel Fabrication

Hybrid hydrogels have been pioneered to help overcome the inherent limitations of both natural and synthetic polymers, which include relatively deficient mechanical properties and immunogenic risks in the case of natural materials and limited biocompatibility and biodegradability in the case of synthetic materials [46].

Hybrid hydrogels exhibit a remarkable performance, combining robust mechanical properties with favorable biocompatibility and degradability. For instance, HA-based injectable hydrogels suffer from mechanical weakness and rapid degradation by endogenous enzymes [107], which lead to mechanical strain and a short in vivo lifespan, limiting their use in load-bearing tissue engineering. Hence, modifications to enhance HA’s properties are imperative to improve its therapeutic efficacy. Similarly, pure collagen hydrogels suffer from insufficient physical strength, prompting the development of hybrid injectable hydrogels comprising collagen and diverse biomaterials [46].

On the other hand, synthetic materials such as PEG lack inherent bioactivity, but this can be enhanced by incorporating bioactive natural materials, thus expanding their application range [108]. Emerging chemistry, including the Schiff base reaction and Diels–Alder reaction, along with various crosslinking methods for different raw materials have facilitated modifications to traditional hydrogels’ properties and enabled numerous new applications. By strategically combining natural and synthetic materials, hybrid hydrogels can leverage their respective strengths and mitigate weaknesses, offering tailored solutions for diverse biomedical applications.

## 4. Types of Hybrid Hydrogels

Hybrid hydrogels encompass various types, each tailored to specific applications, showcasing the adaptability and innovation within hydrogel technology.

### 4.1. Reversible Physical Hydrogels

Reversible physical hydrogels are characterized by a network formation primarily driven by molecular entanglements and secondary forces such as hydrogen bonds, coordination bonds, and electrostatic and hydrophobic interactions [109]. These interactions occur under specific conditions, with physicochemical interactions like stereocomplexation, charge condensation, or supramolecular chemistry crucial in shaping the gel structure [110].

The distinctive characteristic of reversible or physical hydrogels lies in their capacity to shift between a gel state and a homogeneous solution, which is controlled by external factors like temperature, pH, ionic strength, or solvent composition. The reversibility of these hydrogels varies, manifesting diverse stimuli-responsive behaviors. For instance, an injectable, thermosensitive, in-situ gel-forming system comprises a chitosan solution with glycerolphosphate disodium salt (Gp), undergoing a sol–gel transition under specific physiological pH and temperature conditions [111]. Physical crosslinks in these hydrogels can be established through processes like crystallization, involving interactions among amphiphilic block and graft copolymers, as well as protein interactions [109].

Despite their versatility, reversible physical hydrogels are generally unstable and mechanically weak, transitioning from gel to solution under altered conditions and regelling upon returning to the initial environment. Nevertheless, the ability to control their gel–sol transitions through external stimuli renders physical hydrogels valuable for applications where the dynamic responsiveness is a key requirement.

### 4.2. Multifunctional Hybrid Nanogels

**Figure 3:**
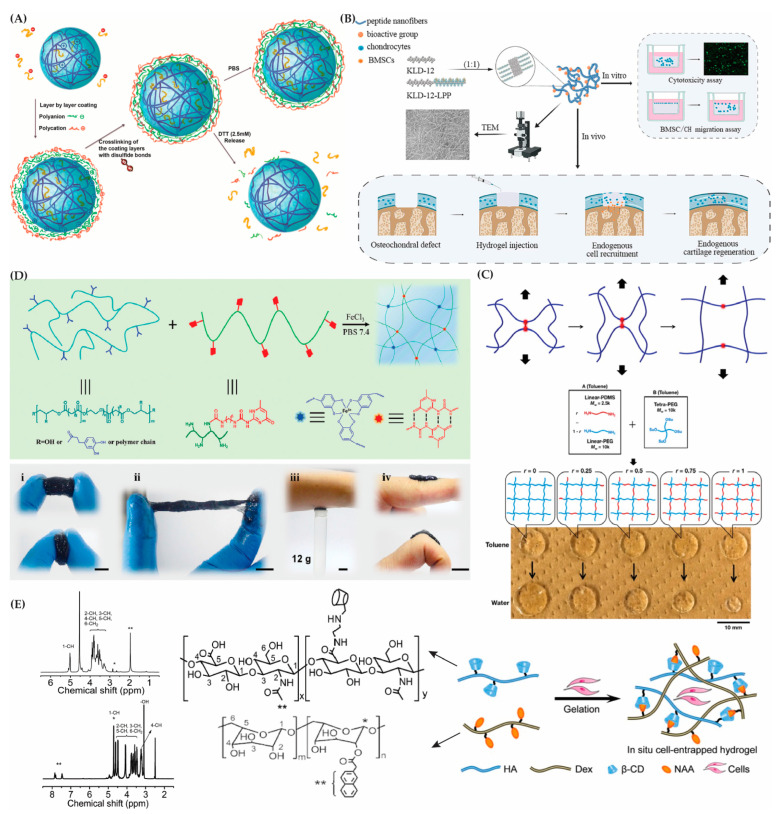
Hybrid hydrogels. (**A**) Layer-by-layer coating process on protein-loaded nanogels. Reproduced with permission from Li et al. [113]. (**B**) Self-assembled peptide hydrogels used with bone marrow mesenchymal stem cells (BMSC) and chondrocytes (CH). Reproduced with permission from Lv et al. [117]. (**C**) Fuse link concept: red lines connect blue network chains, ensuring a constant mechanical profile. Fuse links break under stress (top). Gels with a varying PDMS content are shown in equilibrium-swollen states in toluene and water (bottom). Reproduced with permission from Kondo et al. [121]. (**D**) Preparation of PEGSD/GTU hydrogel. Crafting the hydrogel with a schematic of dual dynamic crosslinking (top panel). Soft hydrogel PEGSD2/GTU5.0 in original and compressed (i), stretched (ii), adhered (iii), and bent (iv) states (scale bar: 1 cm) (bottom panel). Reproduced with permission from Zhao et al. [122]. (**E**) Preparation of supramolecular HA-Dex hydrogel in situ as cell scaffold. Chemical structures and ^1^H NMR spectra of HA-CD and Dex-NAA (7.42–7.76 ppm, Ar H, labeled with **; 4.71 ppm, 1-CH of Dex, labeled with *). Reproduced with permission from Chen et al. [123].

Multifunctional hybrid nanogels with high water contents are gaining attention for their colloidal stability and customizable features. Modified polymers and metals enhance properties like the amphiphilicity and stimuli responsiveness, making them suitable for biomedical applications. An example of a nanogel with unique properties is a mucoadhesive nanogel made from chitosan derivatives (i.e., N-succinyl-chitosan), exhibiting a higher affinity to the mucous epithelium [112]. Another study utilized methacrylated dextran and trimethyl aminoethyl methacrylate (TMAEMA)-based reducing-sensitive nanogels loaded with ovalbumin (OVA) for vaccine delivery, responding to the presence of a reducing agent, enhancing the stability, and promoting antigen presentation (see Figure 3A) [113]. pH-responsive poly(N-isopropylacrylamide) (PNIPAM)-co-acrylic acid nanogels with embedded Au nanoparticles were designed for imaging probes, demonstrating a pH-sensitive behavior and increased drug loading capacity [114]. Overall, these findings emphasize the importance of biocompatible and well-designed nanogels to enhance drug efficacy and minimize side effects.

Despite their advantages, challenges in clinical development include understanding intracellular uptake and addressing clearance by the immune system. Hybrid nanogels with traceability and adaptable properties may aid in studying the biological mechanisms for precise drug delivery. A tunable softness and bioresorbable structures make nanogels key variables in understanding biological responses.

### 4.3. Self-Assembling Hybrid Hydrogels

Self-assembling hybrid biomaterials, formed by combining different classes of molecules, exhibit unique properties and structural organization. In regenerative medicine, self-assembled hybrid hydrogels are gaining attention for their biocompatibility, injectability, and flexibility in loading cells and drugs [115]. These hydrogels, particularly those utilizing self-assembled peptide (SAP) technology, facilitate cell adhesion and migration, making them valuable carriers for stem cells [116].

SAP hydrogels have shown their efficacy in bone regeneration and cartilage repair, exhibiting excellent osteogenic properties. Novel functionalized SAP hydrogels, such the as self-assembling peptide hydrogel (KLD-12/KLD-12-LPP, KLPP) containing link protein N-peptide (LPP), have been developed to enhance cell migration and accelerate cartilage repair (see Figure 3B) [117]. Additionally, SAP hydrogels containing growth factors like VEGF promote angiogenesis, as seen in the RATEA16 hydrogel scaffold [118].

Controlled release mechanisms, including ligand-induced conformational changes and erosion triggered by receptors, offer the precise modulation of drug release from hybrid hydrogels. Receptor-mediated protein release, exemplified by VEGF-loaded hydrogels eroding in the presence of relevant receptors, showcases the versatility of these systems in therapeutic delivery [119]. Hybrid hydrogels self-assembled from an ADNA and PLGA hybrid hydrogel (HDNA) deliver dexamethasone for water-insoluble ophthalmic therapy, promising their application in treating eye diseases [120].

The future development of self-assembled hybrid hydrogels should prioritize biomimetic gel construction, mechanical strength improvement, and controlled degradation. As biomedical applications advance, self-assembled hybrid hydrogels are poised to offer diverse clinical treatment options, especially in tissue engineering and drug delivery.

### 4.4. Chemically (Covalently) Crosslinked Hydrogels

Chemical crosslinking involves the formation of covalent bonds among the polymer chains, resulting in a 3D network structure. Various methods such as radical polymerization, Michael addition, the Schiff base reaction, and epoxy crosslinking are employed for this purpose. These procedures typically require crosslinking agents or the incorporation of functional groups in the polymer chains that are capable of forming covalent bonds. The activation of chemical crosslinking reactions can occur through heat, light, or chemical initiators [124]. For instance, initiators induce the generation of new free radicals on linear polymers under specific conditions like temperature, pH, or radiation in free radical polymerization. Photopolymerization, which is widely used, utilizes ultraviolet or visible light radiation and photoinitiators to initiate polymerization and produce hydrogels [125]. In one study, UV crosslinking was used to create an injectable alginate-based hydrogel incorporating nano-hydroxyapatite (n-HAp) [126], resulting in an enhanced osteogenic potential and mechanical properties, with a further enhancement in the bioactivity through the incorporation of recombinant bone morphogenetic protein-2 (BMP-2).

Although double-network (DN) hydrogels offer promising features, their use of toxic crosslinking agents poses a significant drawback [127]. To address this, a new generation of DN gels with two non-covalent associated networks has been proposed. Kondo et al. (2015) developed a dually crosslinked polymer gel, using a tetra-arm, star-shaped poly(ethylene glycol) (PEG) and poly(dimethylsiloxane) (PDMS) linked by orthogonal cross-coupling [121]. The resulting network, with hydrophilic and hydrophobic components, was evenly distributed through a non-covalent hydrophobic association (see Figure 3C). The strength of this association was adjusted by the molar ratio of PEG-to-PDMS segments. Another innovation involved the preparation of a polymerizable derivative of SA through the catalyzed reaction of 2-aminomethyl acrylate (AEMA) with SA. Under the influence of a 0.05% Irgacure D-2959 initiator, this derivative underwent photopolymerization crosslinking, yielding hydrogels with customizable degradation rates and mechanical strengths [128]. These hydrogels have durable crosslinks, showing excellent compatibility with cells, minimal toxicity, and the capacity to support the health and function of bovine chondrocytes. The inclusion of ester bonds in AEMA improves the biodegradability, enabling the customization of the degradation rates, swelling, and elasticity through alginate modification. Another study introduced a hydrogel adhesive with catechol–Fe^3+^ coordination crosslinked poly(glycerol sebacate)-co-poly(ethylene glycol)-g-catechol and quadruple hydrogen bonding crosslinked ureido-pyrimidinone-modified gelatin (Figure 3D) [122], which demonstrated superior properties such as self-healing, tissue adhesion, and antibacterial activity. In vivo tests displayed improved wound healing over medical glue and sutures.

While chemically crosslinked hydrogels offer excellent mechanical strength, stability, and resistance to dissolution once the crosslinks are formed, their application is constrained due to the involvement of harsh production conditions and toxic chemicals.

### 4.5. Core–Shell Hybrid Polymeric Networks

The core–shell design represents a notable advancement in scaffolding technologies for therapeutic molecules and stem cells. This design consists of distinct inner (core) and outer (shell) components, enabling independent functionalities. Co-concentric nozzle extrusion and microfluidics have successfully produced core–shell nano/microfibers and nano/microspheres [129], facilitating the controlled delivery of signaling molecules and drugs from the core and/or shell to optimize therapeutic effects. Stem cells within the core benefit from isolation, supporting ex vivo culture and in vivo tissue engineering, while encapsulated cells thrive in tissue-like microenvironments, secreting therapeutic molecules to surrounding tissues.

Coaxial electrospun PCL-PLA/hydroxyapatite (HA) scaffolds, designed for bone tissue engineering, feature a high HA content without compromising the structural integrity, whether in 2D sheets or 3D tubes [130]. Various collectors influence fiber characteristics and mechanical properties. The inclusion of HA enhances the bioactivity, BMP-2 release, and cell behavior. Additionally, the PEG encapsulation of BMP-2 in the core of PCL nanofibers enables controlled release [131]. Further advancements employ PLGA and alginate to manage the release profiles in core–shell microcapsules [132]. These composite hybrid scaffolds, synthesized through a scalable approach, exhibit a core–shell structure, with the core providing mechanical support and the shell enhancing the biocompatibility for bone tissue regeneration. They significantly improve the mechanical properties while supporting cell viability, proliferation, and osteogenic differentiation. BMP-2 encapsulation in a PLGA core and vascular endothelial growth factor (VEGF) in a PDLLA shell within core–shell microspheres promoted in vitro osteogenesis and angiogenesis. The sequential BMP-2 and VEGF release enhanced new bone formation in calvaria bone defects, demonstrating sustained BMP2 release and 10-day VEGF release [133].

Despite these advancements, challenges persist in optimizing the biomaterial properties needed for maintaining proper cellular behaviors and ensuring stable cell isolations. Continuous research is crucial for addressing these technological issues and unlocking the full potential of core–shell systems in drug delivery and cell encapsulation.

### 4.6. Interpenetrating Polymer Network (IPN)/Semi-IPN Hydrogels

Interpenetrating polymer networks (IPNs) are intricate combinations of two or more crosslinked polymers, with at least one synthesized or crosslinked in the presence of the others. They are classified into full-IPN and semi-IPN, with the latter incorporating linear polymers into a primary crosslinked network, resulting in faster responses to external stimuli [134]. Semi-IPN hydrogels find applications in regulating molecule release and actuator response speed.

IPN hydrogels exhibit biocompatibility and a high water content [124,135]. Liu et al. fabricated physical IPN hydrogels crosslinked by DNA hybridization and the host–guest recognition of cellulose and cucurbit [8] uril, which was responsive to nuclease and cellulase by the dissolution of one network [136]. Guo et al. documented the creation of IPN hydrogels exhibiting rapid self-healing and antibacterial properties [122]. These hydrogels, composed of poly(glycerol sebacate)-co-poly(ethylene glycol)-g-catechol (PEGSD) and ureido-pyrimidinone-modified gelatin, hold promise for infection treatment and wound healing. Biocompatible IPN hydrogels like BioSIN_x_, comprising GelMA and PEG, show promise in tissue engineering by enhancing cell adhesion and promoting cardiac regeneration [137]. These hydrogels foster a favorable environment for cell adhesion, encapsulation, and enhanced cell growth.

The effective delivery of DOX from PNIPA-dendritic polyglycerol (dPG) semi-IPN nanogels, demonstrated in both in vitro and in vivo models, underscores the importance of the electrostatic interactions in semi-IPN structures [138]. Materials with negative charges displayed a superior loading efficiency, while the in vitro experiments indicated a pH-dependent controlled release profile. The in vivo assays illustrated the semi-IPN nanogel’s ability to overcome tumor cell resistance, resulting in a significant reduction in the tumor volume.

IPN scaffolding systems hold biomedical potential, blending synthetic and natural polymers. Challenges persist in achieving genuine interpenetration, emphasizing bio-compatibility and biodegradability. Natural polymers, especially polysaccharides, are essential for the development of effective IPN systems, as some possess an inherent bioactivity, enhancing the hydrogel’s performance. A focused, systematic approach is crucial for establishing polysaccharide IPN systems as potent tools in drug delivery and tissue regeneration.

### 4.7. Supramolecular Hybrid Hydrogels

Supramolecular hydrogels, formed through non-covalent interactions, provide a versatile platform [139,140]. Their mild formation conditions allow for the direct addition of sensitive molecules, such as proteins, and their dynamic nature facilitates minimally invasive injection. The dense crosslinked network of these hydrogels hinders the diffusion of proteolytic enzymes, protecting bioactive therapeutics from premature degradation [141]. Moreover, their reversible nature allows for repeated release on demand, responding to environmental stimuli.

Chen et al. synthesized a supramolecular hydrogel (HA-CD) by conjugating dextran with 2-naphthylacetic acid and hyaluronic acid with β-cyclodextrin (β-CD) (Figure 3E) [123]. NIH/3T3 fibroblasts thrived in the HA-Dex microenvironment, indicating its potential as a cell scaffold. Dextran modifications, such as carboxymethyl dextran (CMDH) and amino dextran (AD), strengthened hydrogels when combined with a derived C2-phenylalanine gelator (LPF) [142].

Synthetic polymers that are engineered with specific functional groups result in hydrogels with tailored properties. PEG forms supramolecular hydrogels when linked with β-CD and cholesterol [143]. Star-shaped, PEG-based hydrogels serve as effective protein carriers, influenced by the crosslink density for release kinetics. Polymers with a lower critical solution temperature (LCST) enable injectable, thermo-sensitive hydrogels, exemplified by poly(ethylene oxide)-b-poly(propylene oxide)-b-poly(ethylene oxide), poly(vinyl ether), and N-substituted acrylamide polymers like PNIPAm [144]. The addition of acryloyl-β-cyclodextrin to PNIPAm tunes its LCST for controlled release applications [145]. Thermo-sensitive hydrogels utilizing a host–guest interaction, such as pyrene-poly(caprolactone)-b-poly(oligo(ethylene glycol) methacrylate) (Py-PCL-b-POEGMA) with α-CD, exhibit temperature-dependent release profiles crucial for therapeutic use [146].

Overall, supramolecular hydrogels exhibit several advantages as protein delivery systems and for tissue engineering. However, resolving lingering challenges is crucial for successful clinical adoption. Burst release is a prevalent issue in hydrogel systems that necessitates ongoing optimization efforts. Achieving sustained therapeutic protein release over weeks via hydrogels requires innovative molecular engineering to enhance their stability.

## 5. Hybrid Hydrogel Modification Strategies

Hydrogel fabrication employs a variety of strategies, including physical and chemical crosslinking methods. Physical hydrogels rely on environmental triggers such as the temperature, pH, and ionic strength, as well as physicochemical interactions. While they can be formed under mild conditions, physical hydrogels are generally weak and exhibit a poor long-term stability. Chemically crosslinked gels are prepared through methods such as reverse microemulsion, irradiation (UV, electron beam, or gamma radiation), emulsion, radical polymerization/crosslinking, and inverse miniemulsion.

**Figure 4:**
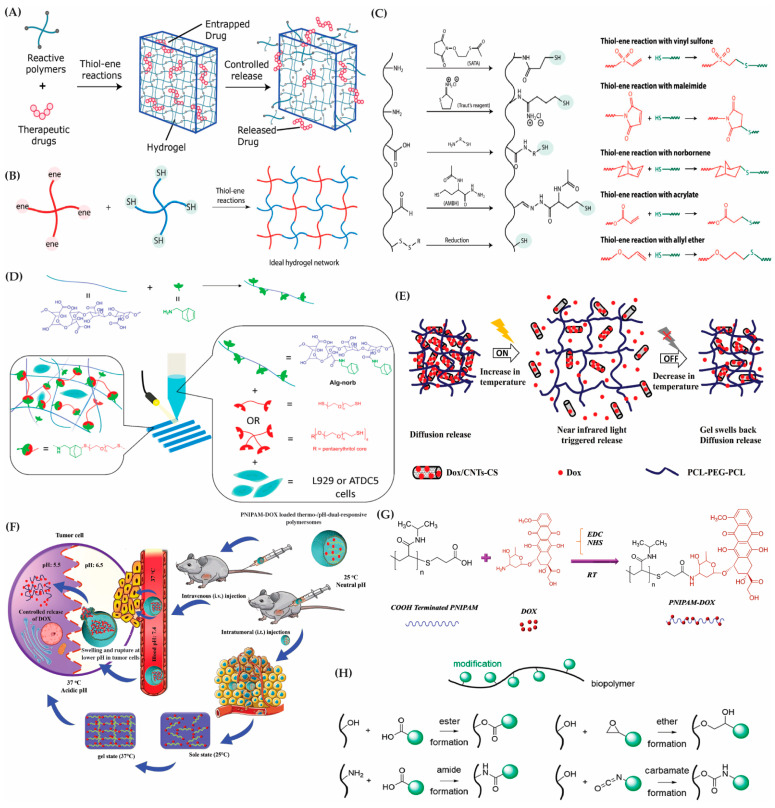
Strategies for hybrid hydrogel synthesis. (**A**) Hydrogel-based delivery vehicles utilize therapeutic loading and release mechanisms. (**B**) Thiol–ene hydrogels form through reactions between alkene and thiol groups on multifunctional macromers. (**C**) Thiol-functionalized macromers react with alkene-functionalized counterparts to create hydrogels for therapeutic delivery, employing various coupling strategies. Reproduced with permission from Kharkar et al. [148]. (**D**) Development of a photoactive alginate bioink (Alg-norb) for 3D bioprinting of hydrogels. Reproduced with permission from Ooi et al. [149]. (**E**) Near-infrared light-triggered drug delivery system using a thermosensitive hydrogel with chitosan carbon. Reproduced with permission from Dong et al. [150]. (**F**) PNIPAM-DOX-loaded polymersomes enable controlled doxorubicin release via temperature and pH responsiveness. (**G**) PNIPAM-DOX conjugate synthesis involves amide bond formation. Reproduced with permission from Oroojalian et al. [151]. (**H**) Introduction of various chemical groups for hydrogel functionalization. Reproduced with permission from Muir et al. [152].

Recent advancements in the chemical transformations of polymers have revolutionized the creation of sophisticated material systems with diverse applications. Notably, the implementation of “click-reactions”, such as a thiol-maleimide Michael addition and thiol-norbornene click reaction, has proven highly advantageous. These reactions offer orthogonality to various naturally occurring chemical functionalities, generate minimal byproducts, and result in a tunable thioether succinimide linkage with reversible and dynamic properties (Figure 4A–C) [147,148].

Click reactions, particularly in hydrogel design, exhibit exceptional biocompatibility, adjustable viscoelasticity, and a unique capacity for responsive cargo delivery, protection, and release to surrounding tissues. This responsiveness can be triggered by biological substrates like glutathione or changes in the pH [153]. For instance, hyaluronic acid hydrogels based on copper(I)-catalyzed azide-alkene cycloaddition (CuAAC) serve as effective cell scaffolds and drug reservoirs [154]. Additionally, the potential toxicity associated with copper-catalyzed reactions can be mitigated through copper-free click chemistries [155]. These chemistries encompass radical-mediated thiol-ene/yne chemistry, tetrazole-alkene photo-click chemistry, the oxime reaction, azide-alkyne cycloaddition, and the Diels–Alder reaction [156].

In hydrogel preparation, both physical and chemical crosslinking methods are employed, with living/controlled radical polymerization techniques such as iodine-mediated polymerization and catalytic atom transfer radical polymerization contributing to the development of versatile and complex structures.

### 5.1. Chemical Modification

Hybrid hydrogels are formed through the covalent crosslinking of the functional groups attached to biopolymers, a process often reliant on catalysts or initiators. The stability conferred by the covalent bonds equips these hydrogels with the potential for long-term stability in both in vitro and in vivo settings. However, the stability of these hydrogels may depend on the network’s degradation capacity. The mechanical and biological properties of the resulting hydrogel are influenced by factors such as the polymer concentration, crosslinking group type, and degree of hybrid hydrogel modification.

Various techniques, including free radical chain polymerization, click chemistry, and phenolic group oxidation, are commonly used to modify hybrid hydrogels. Free radical polymerization involves initiation, propagation, and termination steps, with photoinitiation and redox systems influencing the polymerization rates. Methacrylated hyaluronic acid (MeHA) offers customizable hydrogel properties [157]. The reactive methacrylamide groups in modified hyaluronic acid contribute to free radical polymerization. The addition of diacrylated PEG (PEGDA) enhances the mechanical properties of MeHA hydrogels [158]. GelMA, derived from modified gelatin, proves versatile in forming photopatterned microtissues and microfluidic devices [159]. Hydrolytically degradable groups like esters, which are added to the biopolymer backbone, control hydrogel degradation. Hydroxyethyl methacrylate (HEMA) conjugation to biopolymers, such as dextran and HA, is common for this purpose [152]. The introduction of lactic acid groups accelerates degradation, while caprolactone allows for tunable degradation rates, enhancing neocartilage formation in vitro.

Click chemistry comprises a series of chemical reactions that rapidly form covalent bonds, often in biocompatible settings. These reactions occur in a one-pot system, exhibit a high thermodynamic driving force (greater than 20 kcal/mol), are unaffected by water, demonstrate a high specificity, and generate minimal byproducts [160]. One notable click reaction is thiol–ene radical addition, which forms covalent thioether bonds between alkene and thiol groups, facilitated by radical initiators [161]. This reaction is a powerful tool in biomaterials due to its high yield, mild conditions, regio- and stereospecificity, and biorthogonality. Specifically, thiol–ene reactions involving norbornenes are preferred for biopolymer hydrogel formation, offering superior control compared to other monomers like methacrylates and styrenes [162].

Norbornene, a bridged cyclic hydrocarbon with a strained carbon–carbon double bond, is extensively used in functionalizing biopolymers for thiol-norbornene radical addition crosslinking. Biopolymers such as HA, alginate, cellulose, gelatin, and silk fibroin have been successfully modified with norbornene [152]. For instance, Leuckgen et al. demonstrated that norbornene-modified alginate hydrogels, crosslinked with enzymatically degradable dithiolated linkers, allowed for cell and tissue infiltration in a subcutaneous mouse study [163]. Ooi et al. showcased the use of norbornene-functionalized alginate as a cell-laden bioink for bioprinting tissue engineering scaffolds (Figure 4D) [149]. Moreover, norbornene-modified carboxymethyl cellulose (CMC) underwent thiol–ene crosslinking for cell-laden bioprinting, and cellulose nanofibrils functionalized with norbornene created versatile nanofibril hydrogel suspensions [164]. Combining norbornene-modified silk fibroin with PEG norbornene and DTT produced PEG hydrogels with embedded silk fibroin microgels for potential cellular applications [165]. Apart from norbornene modification, allyl groups have been introduced to biopolymers like gelatin, chitosan, and starch for thiol–ene radical addition crosslinking. Hilderbrand et al. demonstrated the combination of allyl-functionalized collagen mimetic peptides (CMPs) with thiolated PEG to fabricate a photo-cross-linkable hydrogel for 3D cell culture [166].

Yeh et al. devised a photocurable PGS using thiolene click chemistry to control crosslinking [167]. In their approach, a PGS prepolymer (PGSp) was dissolved in anhydrous DCM containing 4-methoxyphenol and DMAP. After purging with nitrogen, 5-norbornene-2-carbonyl chloride dissolved in DCM and triethylamine were added dropwise, and sufficient time was provided to allow the reaction to proceed to completion. DCM was removed by filtration, drying, and rotary evaporation, which yielded a norbornene-modified PGS (Nor-PGS). The crosslinking of Nor-PGS was adjustable by varying the initial PGSp amount. Tsai et al. extended this method by copolymerizing Nor-PGS with PEG, enabling control over the mechanical, degradation, and swelling behaviors of NorPGS-co-PEG [168].

The polycondensation of PGSp with linear polymers such as PCL, PEG, and their acrylated forms methyl ether methacrylate (PEGMEMA), poly(tetramethylene oxide) glycol (PTMO), or gelatin has also been reported [169]. This process entails a two-step procedure, where segments of the linear modifier polymer and sebacic acid polymerize initially. The subsequent addition of glycerol induces the polycondensation of PGS, facilitating the amalgamation of sebacic acid with the linear polymer. By adjusting the content of the modifier polymer and the sebacic acid-to-glycerol ratio, various PGS-co-polymers can be synthesized to attain the desired properties.

Thiol–ene Michael addition crosslinking, which obviates the need for radical initiators, entails reactions between thiols (Michael donors) and electron-deficient -enes (Michael acceptors) [162]. This method, catalyzed by bases or nucleophiles, employs -ene groups like maleimides, vinyl sulfones, acrylates, and methacrylates [170]. The gelation times can be adjusted by varying the -ene group, pH, and biopolymer concentrations. Biopolymers may be functionalized with thiol or -ene groups through esterification or amidation reactions. For example, MeHA crosslinking with DTT exhibited variable gelation times (30 min to 3 h) and sustained erythropoietin release in a rat model [171]. Thiolated HA hydrogels crosslinked with ene-functionalized PEG displayed diverse gelation times (30 s–2 h) and storage moduli ranging from tens to thousands of Pa.

Various modifications of HA, including methacrylates, acrylates, vinyl sulfones, and maleimides, in conjunction with thiolated HA, offer versatile gelation times for applications such as cardiac and neural tissue engineering. Thiolated heparin mixed with PEGDA for Michael addition crosslinking forms a hydrogel suitable for encapsulating and culturing primary hepatocytes [172]. Additionally, thiolated gelatin hydrogels combined with PEGDA hold promise for the rapid encapsulation of MSCs for wound repair [173]. McGann et al. pioneered a microstructured, degradable hybrid hydrogel, melding RLPs and PEG macromers through a Michael-type addition reaction [174]. This involved the reaction of the thiol groups of cysteine residues on the RLP with end-functionalized vinyl sulfone moieties on the four-arm star PEG. The degradation of MMP-specific proteolysis domains, both in solution and within crosslinked hydrogels, was investigated. The RLP24-PEG hydrogels were found to be cytocompatible for the encapsulation of hMSCs. This discovery underscores the hydrogels’ potential for developing structured tissue engineering scaffolds with cell-instructive capabilities, driven by their cytocompatibility, elastomeric properties, microheterogeneity, and degradability.

Azide–alkyne [3 + 2] cycloaddition, known as Huisgen 1,3-dipolar cycloaddition, is another powerful click chemistry tool for forming covalent bonds in a one-pot reaction [175]. For instance, Li et al. developed a thermoresponsive albumin hydrogel using this method [176]. By employing Cu(II) catalysts, the interaction between alkyne-functionalized bovine serum albumin (BSA) and PNIPAm terminated with azide groups resulted in the formation of an azide–alkyne hydrogel. Gelatin hydrogels crosslinked through azide–alkyne reactions with propolic acid-modified lysine residues reached compressive moduli of 50–390 kPa [177]. Copper-catalyzed azide–alkyne cycloaddition reactions were employed for crosslinking HA, cellulose, and alginate [152]. Metal-free azide–alkyne hydrogels were created by functionalizing chitosan with azides, reaching compressive moduli of ∼40–80 kPa within 5–60 min [178]. Elastic-like proteins (ELPs) functionalized with azide or bicyclononyne moieties underwent SPAAC crosslinking, forming hydrogels within minutes for encapsulating cells with a high viability and phenotypic maintenance [179].

In addition to chemical modifications, hybrid hydrogels can be created by grafting synthetic polymers onto biopolymer backbones, thereby expanding the range of functionalities. PNIPAm, for example, has been integrated into various biopolymers like alginate, HA, CS, chitosan, silk fibroin, and gelatin, resulting in thermoresponsive hydrogels [152]. Thiol–ene radical addition grafts PNIPAm onto keratin, creating a thermosensitive keratin-g-PNIPAm hydrogel [180]. Amidation between PNIPAm-COOH and aminated HA yielded injectable AHA-g-PNIPAm hydrogels for adipose tissue engineering [181]. Chitosan functionalized with PNIPAm, forming chitosan-g-PNIPAm hydrogels, serves as an injectable vehicle for antiglaucoma drug delivery in a rabbit glaucoma model [182].

### 5.2. Functionalization

Surface functionalization involves modifying the outer layers of hybrid hydrogels, imparting specific functionalities critical for tailoring the properties of the hydrogels to meet the performance requirements for drug delivery and tissue engineering.

In a notable study, hollow Ca–Alg/PAA hydrogel beads were successfully crafted through UV polymerization, with PAA playing a crucial role in reinforcing the gel structure [183]. The pH-sensitive swelling behavior of these beads is advantageous for drug delivery into the gastrointestinal tract. Furthermore, the cell response to these hydrogel beads indicated their biological safety.

Branched PEG-based copolymers, particularly those derived from POEGMA, provide a versatile approach to modifying PEG hydrogel surfaces. These copolymers, featuring a methacrylate backbone and tunable-length PEG side chains, allow for easy free radical polymerization. The effectiveness of thermoresponsive oligo(ethylene glycol)-based gold surfaces in controlling cell adhesion within a practical temperature range has been demonstrated, presenting new possibilities for advanced functional surfaces in cell culture, bioseparation, and diagnostics applications [184].

Layer-by-layer (LbL) hydrogels can be modified with polymers to influence their mechanical properties and surface charge, which is crucial for cell adhesion [185]. Nanoscale functionalized LbL hydrogels, made of sodium alginate and gelatin, displayed enhanced cartilage healing [186]. The addition of genipin improved the water stability and compression strength, while gelatin coating and LbL assembly with specific peptides enhanced the chondroblast activity and glycosaminoglycan secretion at the nanoscale.

Inspired by carnivorous plants and desert beetles, medical implants featuring a bilayer coating of tethered and mobile perfluorodecalin were designed to mimic slippery surfaces, preventing biofouling in pig arteriovenous shunts [187]. This concept has been extended to skin moisturization by combining slippery nanocoatings with surface bumps, which facilitates their use in low-friction environments [188].

### 5.3. Stealth Functionalization

Hybrid hydrogels designed for biomedical applications require a primary focus on the optimal biocompatibility to reduce the immune response. Simultaneously, enhancing the blood supply, drug biodistribution, and bioavailability is essential. Meeting these criteria requires the meticulous construction of hybrid nanogels, a task that entails specific targeting and molecular designs.

The versatility of hybrid nanogels becomes apparent through a broad spectrum of architectures, achieved through modification, functionalization, and decoration [189]. Their adaptability extends to conjugation with both inorganic and organic nanostructures, offering a vast array of morphologies dependent on assembly techniques, particle forms, and architectural variations in the size and core–shell composition [190,191]. The attainment of diverse morphologies can be attributed to physical crosslinking or chemical reactions, mediated by ionic interactions, hydrogen bonds, and other intermolecular forces.

Critical parameters influencing biodistribution include appropriate biocompatibility and surface decoration. The components, surface charge, and ligand interactions also play pivotal roles. Covert functionalization, exemplified by polyethylene glycols or chitosan, manipulates hydrophilic polymeric chains for stealth purposes. Thus, the intricate interplay of these factors defines the success of hybrid nanogels in biomedical applications, emphasizing the need for precision in their design and functionalization.

### 5.4. PEGylation

PEGylation, the process of attaching PEG chains to nanostructures, extends the circulation lifespan and enhances drug bioavailability. Such modification forms a protein corona, limiting plasma protein enclosure and macrophage absorption [192]. The effectiveness of PEGylation depends on factors such as the hydrophilic characteristics and molecular weight of the PEG chains, which typically range from 2 to 13 kDa.

In PEG-hydrogel drug delivery systems (DDSs), drugs are typically encapsulated through non-covalent entrapment, preventing chemical bonding and allowing their gradual release as the responsive segments break. PEG’s in vivo metabolism and biosafety make it a desirable biomaterial for DDSs. Incorporating imaging agents into PEG-hydrogel nanomedicine allows for therapy visualization [193]. Recent studies showcase innovative applications of PEG-based hydrogels. For instance, Lv et al. designed a thermosensitive hydrogel system with a four-arm PEG–PCL copolymer, incorporating porphyrin for fluorescence imaging [194]. Another study by Lv et al. developed a programmed PCL–PEG–PCL thermosensitive hydrogel combined with chitosan-multiwalled carbon nanotubes and model drugs for real-time tracking through in vivo fluorescence imaging [195]. In another study, Dong et al. innovated a thermoresponsive drug delivery system using a PCL–PEG–PCL hydrogel with chitosan-multiwalled carbon nanotubes (Figure 4E) [150]. This setup offers a dual-stage drug release and enables its controlled delivery through near-infrared light activation, enhancing sustained therapeutic effects.

PEG has found widespread use in chemical crosslinking and end-capping treatments for pH-responsive hydrogels. Methacrylic acid (MAA) is commonly employed due to its biocompatibility. Ahmad et al. utilized MAA in a chemically crosslinked PEG-poly(methacrylic acid) oral hydrogel for the pH-responsive, colon-targeted administration of oxaliplatin (OXP) [196]. Multi-stimuli-responsive hydrogels, combining the pH with thermal, magnetic, redox, or photothermal responses, address the limitations of DDSs. An example is a thermo-pH responsive DDS with PNIPAm-DOX loaded into a pH-responsive polyethylene glycol-2,4,6-trimethoxybenzylidene pentaerythritol carbonate (PEG-PTMBPEC) polymer, significantly enhancing the therapeutic effects in vivo (Figure 4F,G) [151].

PEG-based hydrogels have shown promise in diverse biomedical applications. Hydroxyapatite nanoparticles in PEG hydrogels enhance the alkaline phosphatase activity, mineral deposition, and collagen type I formation, making RGD sequence-attached, PEG-based, MMP-sensitive hydrogels promising for bone tissue engineering [197]. Hockaday et al. designed 3D-printed PEG-DA aortic valve scaffolds that mimic native tissue, showing an increased elastic modulus and tissue-specific engraftment [198]. PEG-based hydrogels, including PEGylated dendrimer nanocarriers, demonstrated clinical potential for cartilage tissue repair and the enhanced delivery of biological drugs in osteoarthritis treatment, as evidenced by the improved pharmacokinetics and efficacy in a rat model [199].

Due to their low cytotoxicity, biocompatibility, drug-encapsulating capability, circulation stability, and tunable mechanical properties, PEGylated hydrogels hold great promise in various biomedical applications, including DDSs, tissue engineering, gene therapy, wound healing, and antibacterial scaffolds.

## 6. Nano/Microstructure Incorporation into Hybrid Hydrogels

The integration of nano/microstructures into hydrogel formulations enables the creation of hybrid hydrogels with diverse functionalities for use in biological systems. This incorporation of particles and the formation of domains not only facilitate targeted drug therapy and tuned cellular responses but also result in a stimuli-responsive material behavior, along with enhanced mechanical and physical properties. As the evolution of hybrid hydrogels progresses, the design of new systems will draw inspiration from biological structures, mirroring the intended environment. Significantly, easily synthesized one-step methods and modular tunability will be influential in advancing these hybrid hydrogels for specific applications.

### 6.1. Hybrid Hydrogels with Integrated Nanostructures

Composite nanoparticle–hydrogel structures can be formed through non-covalent or covalent immobilization methods, utilizing various nanoparticle types and hydrogel frameworks [28]. To achieve a uniform distribution of nanoparticles in hydrogels, several strategies are employed, including direct hydrogel formation in a nanoparticle suspension, embedding nanoparticles post-gelation, reactive nanoparticle formation within preformed gels, nanoparticle-mediated crosslinking, and gel formation through crosslinking a mixture of nanoparticles, polymers, and gelator molecules [28]. Notable advancements in hydrogel mechanical properties include silica nanoparticles increasing the stiffness, nitro-dopamine-modified iron oxide nanoparticles leading to a 10-fold stiffness rise in collagen-based hydrogels, carbon nanotubes tripling the tensile modulus in gelatin hydrogels, and gold nanoparticles elevating the shear modulus 6-fold in PNIPAm hydrogels [200,201,202].

Conventional hydrogels face limitations in effectively encapsulating hydrophobic therapeutic molecules, prompting the development of alternative strategies for controlled drug release [203]. For instance, the direct loading of the hydrophobic drug dexamethasone acetate (DMSA) into poly-2-hydroxyethyl methacrylate (pHEMA)-based hydrogels resulted in only a three-day release. However, incorporating DMSA-loaded poly(ethylene glycol)-poly(ε-caprolactone) (PEG-PCL) micelles into the hydrogels achieved sustained release for up to 30 days [204]. Other studies explored the encapsulation of hydrophobic drugs like erythromycin and curcumin in micelles and nanoparticles incorporated into hydrogels, achieving their controlled release for specific durations [205,206].

**Figure 5:**
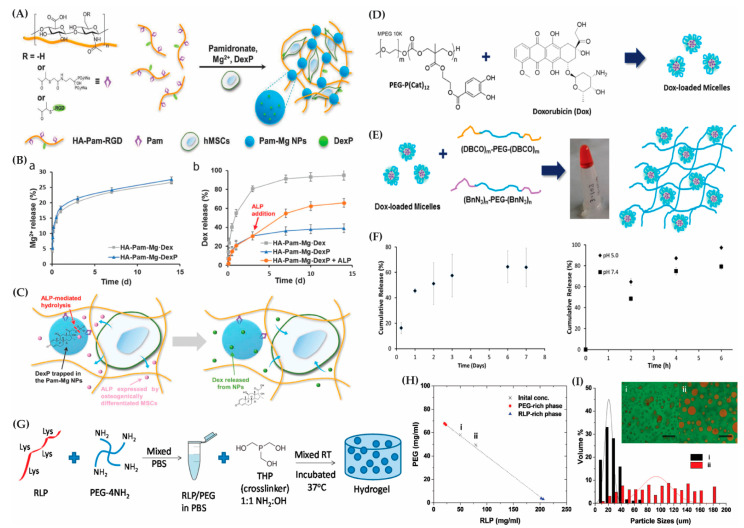
Different nano/microstructure incorporations in hybrid hydrogels. (**A**) Fabrication of self-assembled HA–Pam–Mg nanocomposite hydrogels. (**B**) Sustained release of Mg^2+^ (a) and Dex (b) from HA–Pam–Mg hydrogels, with variations in the Dex loading methods and cumulative releases in PBS buffer. (**C**) Biomarker-responsive Dex release from HA–Pam–Mg–DexP hydrogels through a positive feedback circuit. Reproduced with permission from Zhang et al. [209]. (**D**) DOX (doxorubicin) encapsulated using PEG-P(Cat)_12_ and the formation of DOX-loaded micelles. (**E**) Hydrogels loaded with DOX prepared by SPAAC crosslinking, incorporating DOX-loaded micelles. (**F**) Release of DOX-loaded micelle/hydrogel composite in PBS (pH 7.4) at 37 °C, and DOX-loaded micelle solutions in PBS (pH 7.4) and acetate buffer (pH 5.0). Error bars represent the average ± standard deviation. Reproduced with permission from Ono et al. [210]. (**G**) Crosslinking reaction between THP and primary amines, stabilizing RLP-rich and PEG-rich domains in the hydrogel network. (**H**) RLP and PEG concentrations. (**I**) Particle size distribution and confocal images of hydrogel. Reproduced with permission from Lau et al. [211].

Stimuli-responsive hybrid hydrogels have been designed for chemotherapeutic applications, employing chemical and biochemical approaches for hydrogel degradation and drug release triggered by specific stimuli. Liposome-crosslinked hybrid hydrogels responsive to thiol-containing environments were developed for controlled doxorubicin release [207]. Additionally, a bioactive nanocomposite hydrogel, formed by hyaluronic acid and self-assembled pamidronate-magnesium nanoparticles, enabled the localized elution and simultaneous release of bioactive ions and a small drug for bone regeneration [208]. Another study by Zhang et al. introduces an HA-based bioactive nanocomposite hydrogel incorporating self-assembled pamidronate-magnesium nanoparticles (HA–Pam–Mg) [209]. This hydrogel enables the localized elution and simultaneous release of bioactive ions and small molecule drugs (Figure 5A–C). Its excellent injectability and stress relaxation facilitates its easy adaptation to irregular bone defects, while the released magnesium ions promote the osteogenic differentiation of encapsulated human mesenchymal stem cells, triggering a positive feedback loop that significantly enhances bone regeneration.

In another example, biodegradable, polycarbonate-based ABA triblock copolymers were chemically crosslinked into hydrogels, encapsulating doxorubicin-loaded, catechol-functionalized polycarbonate micelles (Figure 5D,E) [210]. This formulation sustained doxorubicin release for a week, exhibiting a pH-dependent profile with an increased release at a lower pH, impacting the viability of MDA-MB-231 breast cancer cells (Figure 5F). The development of these chemically degradable hybrid hydrogels showcases their potential as platforms for the controlled and targeted delivery of various therapeutic cargoes. The diverse approaches presented, ranging from micelle incorporation to stimuli-responsive formulations, highlight the versatility of hybrid hydrogels in addressing the challenges of controlled drug release for both hydrophilic and hydrophobic compounds.

Hydrogels with nanoscale structures have been explored to modulate cell behaviors by optimizing their mechanical properties or improving their stability. While highly crosslinked 3D hydrogels may limit cell activities, incorporating nanoparticles like carbon-based structures, dendrimers, and ceramics can create biomimetic 3D environments. Carbon-based nanostructures enhance hydrogel mechanical properties, promoting cell proliferation and differentiation, resembling native tissues such as bone and muscle [212]. Carbon nanotube–gelatin methacrylate hydrogels significantly improve the compressive modulus and cell survival [201]. Hybrid hydrogels with primary amine-terminated polyamidoamine dendrimers enhance cell proliferation in collagen scaffolds, showcasing an increased biostability. These innovations hold promise for regenerative medicine and drug discovery by mimicking the native tissue complexity.

### 6.2. Incorporation of Metallic Nanoparticles in Hybrid Hydrogels

Due to their antimicrobial properties, as well as their effects on surface and electrical properties, a combination of noble metal nanoparticles (NPs) and hydrogels is actively being investigated. Recent studies have tested thermosensitive chitosan/phosphate hydrogel composites containing silver (Ag) and Ag–palladium core–shell NPs with various cell lines, including skin fibroblasts, hepatocellular carcinoma, and breast cancer cells [213]. These composites exhibited an exceptional cell viability. Zulkifli et al. conducted a study on antimicrobial hydroxyethyl cellulose–Ag NP scaffolds, which notably promoted the growth and proliferation of human fibroblasts, possibly due to an increased surface roughness, enhancing cell adhesion and proliferation [214]. González-Sánchez et al. demonstrated the synthesis of Ag NP-based methacrylate hydrogels, showcasing excellent biocompatibility with osteoblast cells and antibacterial activity [215]. Qiu et al. reported on the synthesis and characterization of a nanocomposite hydrogel dressing, HMC-HA/AgNPs, which displayed potent antibacterial activities against *Staphylococcus aureus* and *Escherichia coli* [216]. This dressing effectively repaired wound defects in mouse models, achieving significant wound healing within a short period.

Additionally, a silk fibroin/nanohydroxyapatite hydrogel modified with Ag and Au NPs showed an enhanced mechanical stiffness, facilitating the attachment and spreading of osteoblast cells. In cardiac tissue engineering, incorporating Ag and gold (Au) NPs into hydrogel composites has been explored to enhance the electrical properties. For instance, the electroactive Au NP-impregnated thiol 2-hydroxyethyl methacrylate (HEMA)/HEMA composite hydrogel showed an enhanced expression of connexin-43 in cultured cardiomyocytes [217]. Moreover, Au NPs deposited on decellularized matrices resulted in stronger contraction forces and faster calcium transients in neonatal rat cardiomyocytes.

Despite these advancements, concerns persist regarding the cytotoxicity and release of metal NPs from the scaffolds. The interaction between NPs and cells remains poorly understood, necessitating thorough cytotoxicity studies before advancing to in vivo models. While entrapping NPs within the hydrogel structure can mitigate massive NP uptake by cells, the risk of uncontrolled NP release remains a concern. Therefore, the careful consideration of biocompatibility and the controlled release mechanisms is imperative in the development of NP–hydrogel composites for biomedical applications.

### 6.3. Hybrid Hydrogels with Integrated Microstructures

The incorporation of micron-sized particles or domains into hydrogels enhances the structural integrity, providing a means to influence cell behaviors. Cells respond to material properties such as the elasticity and pore size, making it advantageous to exploit these properties for developing materials that stimulate cell growth, proliferation, and migration [218]. Hydrogels commonly used for drug release and cell culture lack the microstructural diversity found in the ECM and tissues. Inspired by natural microstructures, hybrid hydrogels with micron-sized domains have been engineered to support cellular growth, enable microstructure-mediated cell migration, and facilitate the controlled release of bioactive agents.

Microparticles can be covalently or non-covalently incorporated into a hydrogel network to yield an increased mechanical strength, release bioactive molecules, and promote desirable cellular responses. A study explored enhancing hydrogel mechanical properties by incorporating microparticles, focusing on graphene derivatives (GDs) in peptide nanofiber matrices [219]. Three β-sheet-forming peptides and five GDs with diverse surface chemistries were examined to understand the impacts of electrostatic and hydrophobic interactions on the hydrogel’s modulus. When both types of interactions were attractive, the storage modulus (G′) increased, while the net effect on G′ under competing interactions was dependent on their relative strengths.

Hybrid hydrogels release bioactive molecules through three mechanisms: burst release from surface-dissolved drugs, diffusion through the hydrogel matrix or microparticles, and release during hydrogel degradation [220]. These mechanisms allow for the modulation of transport, with polymer network and particle composition adjustments controlling the mesh size and degradation rates. Introducing bio-responsive domains enables cell-responsive behaviors. For instance, vancomycin hydrochloride HMPC nanoparticles in a chitosan/glycerophosphate hydrogel extended drug release through diffusional transport [220]. Tailoring drug release in a carbohydrate-based injectable hydrogel (PNIPAm functionalized with acrylic acid) involved anionically functionalized microgels, controlling the release through diffusive contributions and drug partitioning between bulk and microgel phases [221]. However, tuning release within micro-domains is limited by geometry, as larger particle diameters restrict diffusion. To address this, spheroid particles with an increased surface area have been generated, showing a faster release of encapsulated BSA compared to spheres.

Anisotropic microparticles, such as spheroids, ellipsoids, rods, and disks, have demonstrated significant benefits in hemodynamic settings, showing improved margination, wall interactions, and adhesion [222]. The incorporation of spheroid particles has been linked to enhanced diffusion and cell viability. Anisotropic particles in hybrid hydrogels also exhibit potential hemodynamic transport advantages. Non-circular micropatterned regions in hybrid hydrogels enhance toxin absorption, thereby increasing the detoxification efficacy. Star-shaped channels in a PEGDA/red blood cell membrane-coated nanoparticles hybrid hydrogel demonstrated a higher detoxification efficacy compared to circular channels, likely due to the increased surface perimeter and multiple planes [223].

Beyond microparticle incorporation, various methods, such as liquid–liquid phase separation, emulsion stabilization, and photopatterning, have been used to produce hydrogels with micron-sized domains. Among these, phase separation offers one-step fabrication, tunability, and specific behavior. Resilin-like polypeptide (RLP)–PEG hydrogels, created through liquid–liquid phase separation and UV-crosslinking, feature RLP-rich microdomains within a PEG matrix (Figure 5G,H) [211]. These hydrogels support a high cell viability and allow for direct cell localization around RLP-rich domains. The domain size can be adjusted by controlling the timepoint of crosslinking during phase separation (Figure 5I). While this method offers in situ applications and tunability in the domain size, it has limitations in the range of structures achievable. Overall, anisotropic microparticles and phase separation techniques showcase potential advancements in designing hybrid hydrogels with improved hemodynamic properties and detoxification efficacies.

Laser-mediated UV photopatterning has been proposed to enhance the precision and efficiency of hydrogel manufacturing. Traditional methods, such as 3D printing and photopatterning, are limited in resolution and are labor intensive [224]. Laser-mediated UV photopatterning proves less time consuming and highly precise, addressing these limitations [225]. For instance, using riboflavin-50 phosphate gelatin hydrogels as a non-toxic UV photosensitizer accelerates the development of micropatterned substrates with an improved spatial resolution [226]. Combining this with the crosslinking and casting of polymeric materials allows for the creation of hybrid hydrogels with finely tunable domains. The resulting micropatterned gels can be assembled into multilayered materials with high-resolution microchannels for improved perfusion, exemplified by a hollow microchannel hydrogel that facilitates faster cell viability over 7 days.

## 7. Mechanisms for Bioactive Molecule Release from Hybrid Hydrogels

Efficient delivery systems play a crucial role in maximizing therapeutic outcomes while minimizing side effects. One common challenge in drug delivery is the burst release phenomenon when bioactive molecules are dispersed within polymer networks without proper control. To address this issue, various strategies involving polymer particle-based systems and hybrid hydrogels have been explored, offering controlled, long-term release and customization in terms of the size, composition, and functionality [124].

The integration of polymer particles into hydrogels has led to the development of hybrid or “plum pudding” systems [110]. These systems have demonstrated the ability to delay the release of bioactive molecules such as proteins. For example, TGF-β1 loaded into PLGA microspheres embedded in PEG gels exhibited reduced burst release compared to microspheres alone [227]. The cumulative release was further delayed with an increasing microsphere concentration. These hybrid systems create barriers for diffusion, reducing the burst release effects and enhancing biocompatibility by concealing particles within the polymer networks.

To overcome the rapid depletion of hydrophilic and small molecule drugs from hydrogels, encapsulation into polymer microparticles before incorporating them into hydrogels has been proposed. This approach has been successful in achieving the sustained release of various drugs, including lidocaine, bupivacaine, and DOX. To counter the swift depletion of hydrophilic and small molecule drugs from hydrogels, a viable approach involves encapsulating these drugs within polymer microparticles before their integration into hydrogels. For example, lidocaine, which exhibits rapid release from poloxamer hydrogels within one day, showcases prolonged release over two days when formulated as a microsphere–poloxamer system [228]. Similarly, anionic microspheres within DEX/CMC hydrogels achieve the sustained release of bupivacaine [229]. Various poly-acid anionic polymers such as PAA and PMAA, as well as cationic polymers like chitosan and polyhistidine, have been investigated for their pH-sensitive properties, influencing the release modulation in diverse drug delivery systems [230].

pH-sensitive micelles act as carriers for bioactive molecules, and their release mechanisms can be categorized into four types: (i) polymer protonation or deprotonation, (ii) the hydrolysis of acid-sensitive bonds within the polymer, (iii) alteration of hydrophobicity in polymer micelles, and (iv) disruption of acid-sensitive bonds between bioactive molecules and carriers [231]. Bioactive molecules are loaded through covalent bonds or by physical attachment in carriers possessing a crosslinked matrix or self-assembled structure [232]. Enzymatic release involves the hydrolysis of enzyme-sensitive polymers, cleavage of enzyme-sensitive linkers, and disruption of enzyme-sensitive bonds [233].

Temperature-responsive polymers, known for their ability to release bioactive substances in response to temperature fluctuations facilitated by polymer chain expansion, play a crucial role in applications such as gene delivery and tissue engineering. Hydrogels exhibit varying degrees of swelling in reaction to temperature changes. Those with an LCST or upper critical solution temperature (UCST) close to body temperature are particularly suitable for biomedical applications [234]. An illustrative example involves the utilization of PNIPAm hydrogels as carriers for antimicrobial agents, wherein a thermoresponsive hydrogel with PLLA- and dextran-cleavable groups demonstrated temperature-dependent swelling and degradation [235]. Bovine skin gelatin gels containing polystyrene nanoparticles were combined with a PNIPAm network to improve the mechanical characteristics and thermoresponsive efficiency of the hybrid composite [236]. In a separate study, chitosan was modified with PNIPAm using the sonication technique, resulting in the formation of a thermoresponsive hydrogel [237]. The resulting nanogels could deliver curcumin to tested cells (MDA-231, Caco-2, HepG2, and HT-29 cancer cell lines) and exhibited a dose-dependent cytotoxicity against cancer cells.

IPN hydrogels sensitive to temperature have been developed for bioactive release. For instance, Shin et al. fabricated an IPN hydrogel from PAA and PVA, swelling at temperatures higher than its UCST due to hydrogen bonding disruption, leading to bioactive release [238]. Additionally, Wang et al. synthesized a thermoresponsive IPN using polyacrylamide and PAA-graft-β-CD, exhibiting swelling at temperatures beyond its UCST and resulting in rapid bioactive release [239].

An alternative approach to induce the release of encapsulated bioactive molecules from nanocarriers using light involves incorporating photo-switchable groups, such as azobenzene, into the nanocarrier shell pores. Azobenzene-doped liposomes encapsulating phospholipid-modified upconverting nanoparticles (UCNPs) and the anticancer drug DOX were utilized in this context. The conversion of near-infrared (NIR) light into ultraviolet (UV) and visible light facilitates the photoisomerization of azobenzene amphiphiles, leading to the release of the encapsulated drug [240]. In a study by Peng and colleagues, they developed a dextran-based, photo-responsive hydrogel system [241]. Utilizing the inclusion complex of CDs and trans-azobenzene in their formulation acted as a photo-switchable crosslinker. Upon UV irradiation, trans-azobenzene transformed into cis-azobenzene, disrupting the network formed by CDs and dextran, transforming the hydrogel into a sol, and facilitating the release of bioactive molecules.

For the targeted delivery of anticancer agents to tumor cells, redox/enzyme and redox/pH-responsive carriers are also advantageous [242,243]. Specifically, self-assembled nanoparticles featuring redox-sensitive bonds can release docetaxel triggered by hyal-1 and glutathione, leveraging lysosomal enzyme hydrolysis and reducing the agent degradation of disulfide bonds [244]. These tailored carriers enhance targeted drug delivery, promising improved therapeutic outcomes.

Advancements in controlled release systems, including hybrid hydrogels and responsive polymers, have addressed challenges associated with burst release and enabled the precise modulation of drug release rates. These innovative strategies offer potential applications in various fields, from conventional drug delivery to emerging areas like gene therapy and cancer treatment. The ongoing research in this field holds promise for further improving drug delivery efficiency and therapeutic outcomes.

## 8. Encapsulation of Cells and Biomolecules within Hybrid Hydrogels

Hydrogels provide an optimal delivery vehicle for a spectrum of biological agents, from simple molecules to cell clusters, facilitating diverse therapeutic strategies. Stimuli-responsive polymers, in particular, allow for lightly crosslinked hydrogels that can encapsulate biomolecules or cells and are able to release their cargo in a controlled way for minimally invasive and localized drug or cell therapies.

### 8.1. Encapsulation of Biomolecules

The utility of hydrogels for the encapsulation and controlled release of proteins has been demonstrated in multiple studies, including the delivery of growth factors for enhancing wound healing, bone morphogenetic protein 2 (BMP-2) for bone regeneration, insulin for oral delivery or triggered release, and diverse antigens and antibodies for immunotherapy [245,246,247,248,249,250,251]. An unresolved issue remains the phenomenon known as burst release. This is a sudden release that can be attributed to proteins weakly bound to the hydrogel or those located near its periphery. Burst release have been shown to range from 25% to nearly 50%, depending on the hydrogel composition [252].

Hydrogels have also found application in the delivery of growth factors. Regenerative medicine often relies on stem cells and potent growth factors at high doses, which are expensive and pose risks if delivered into the non-targeted tissues. Ma et al. reported the use of therapeutic small extracellular vesicles (t-sEVs) loaded with human VEGF-A and BMP-2 mRNAs [253]. These t-sEVs, enclosed in a tailored injectable hydrogel, stimulated bone regeneration in rats with femur defects. Produced via nano-electroporation, t-sEVs can deliver plasmid DNAs to human adipose-derived mesenchymal stem cells (hAdMSCs), promoting angiogenic and osteogenic regeneration. Controlled release within the hydrogel confines treatment to the defect site, fostering efficient bone regeneration with minimal systemic effects.

Viruses can also be delivered using hydrogels. While many viruses are associated with diseases, some can serve beneficial purposes, including bacteriophages used as alternatives to antibiotics and vectors for delivering nucleic acids in genetic engineering. Hydrogel encapsulation protects the viruses from the environment and from immune recognition and allows their release on the targeted sites. For instance, in phage therapy, oral administration is convenient but faces multiple challenges, including acidic inactivation in the stomach [254]. Hydrogel encapsulation can protect phages and facilitate their release in the small intestine, improving their therapeutic efficacy [255].

### 8.2. Encapsulation of Cells

Encapsulating cells within hydrogels holds significant promise for multiple applications. Besides providing a protective shield and a conveyance method, hydrogels can also create an environment conducive to cell differentiation or phenotype maintenance. In these cases, it is critical to consider the interactions between the hydrogel and the cells being carried, as together, they constitute the cell therapy system.

Hydrogel systems have been developed for the delivery of probiotics, protecting the probiotic bacteria from the stomach acid, ensuring their timely release in the intestine, and promoting colonization [256]. For instance, hybrid xanthan–chitosan hydrogel formulations have demonstrated excellent performance in maintaining probiotic viability and controlled release under simulated gastrointestinal conditions [257].

In cell transplantation therapies, hybrid hydrogels aid in shielding cells against immune recognition while permitting essential nutrient and molecule passage [258]. For instance, encapsulating islets within photopolymerized PEG-DA hydrogels has demonstrated success in maintaining normoglycemia in diabetic-induced rats for an extended period, indicating a promising avenue for treating insulin-dependent diabetes [259].

Hybrid hydrogel-encapsulating chondrocytes have also been used for forming cartilage. The ability of alginate to induce the redifferentiation of chondrocytes, which have been previously dedifferentiated through monolayer cell culture, has been demonstrated. Moreover, alginate/hyaluronan hybrid hydrogels loaded with TGF-β 1 were shown to facilitate the differentiation of various stem cells into a chondrogenic phenotype. Differentiation was shown to be influenced by the hydrogel’s elasticity modulus [260]. The similarity in the mechanical properties between the hydrogel and cartilage aids in directing cell differentiation toward a chondrogenic lineage.

Utilizing photopolymerization reactions enables the swift creation of hydrogels in situ under conditions conducive to cell viability, facilitating minimally invasive cell-based therapies. Photopolymerized, PEG-based hydrogels for cartilage tissue engineering have demonstrated the successful encapsulation, viability maintenance, and differentiation of chondrocytes. These hydrogels exhibited homogeneity in cell and extracellular matrix distribution, mimicking native cartilage tissue [21]. The ability to form hydrogels in situ, under mild conditions, that are compatible with biomolecules and cells, have also been favored in hard tissue applications, such as bone healing and orthopedic treatments. Incorporating BMP-2 within PEG, alginate, or elastin-like peptide hydrogels has stimulated osteoblast activity and enhanced bone regeneration both in vitro and in vivo [261].

Zwitterionic hydrogels are attractive for cartilage tissue engineering due to their hydrophilicity, lack of immunogenicity, and antifouling properties. Yet, their use is hindered by the lack of injectable variants for cell encapsulation. To overcome this constraint, Asadikorayem et al. introduced injectable, self-healing zwitterionic granular hydrogels, enabling direct cell encapsulation for cartilage engineering [262]. This method involves the mechanical fragmentation of bulk photo-crosslinked hydrogels containing carboxybetaine acrylamide (CBAA) or a blend with zwitterionic sulfobetaine methacrylate (SBMA). Encapsulated human primary chondrocytes thrive within this matrix, displaying a high viability, proliferation, migration, and cartilaginous ECM production. Such techniques offer a scalable method to broaden the utility of zwitterionic hydrogels in tissue engineering.

## 9. Biomedical Applications of Hybrid Hydrogels

The versatility of hybrid hydrogels makes them promising materials for applications in drug delivery, tissue engineering, diagnostics, and medical coatings. When employed as drug carriers, they enable precise therapeutic release with adjustable kinetics, responding to environmental cues to provide on-demand precision. In tissue engineering, these hydrogels serve as scaffolds, creating a conducive three-dimensional environment that supports cell adhesion, proliferation, and differentiation. Due to their adaptability and versatility, these materials show great promise in addressing diverse challenges in medicine and enhancing patient outcomes. These applications are summarized in Table 1.

### 9.1. Hybrid Hydrogels as Drug Delivery Systems (DDSs)

**Figure 6:**
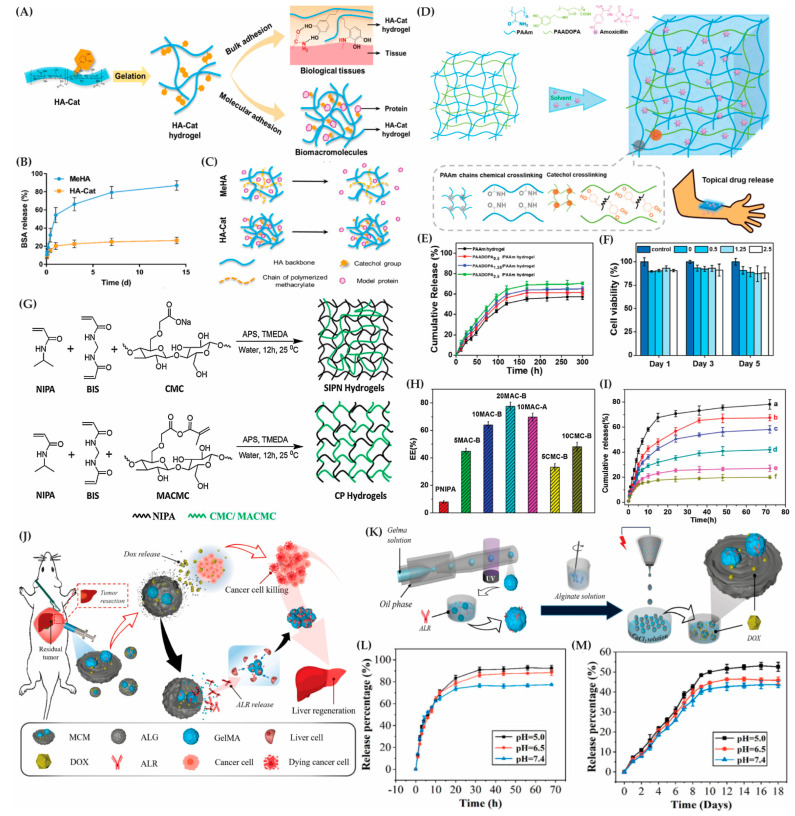
Drug delivery systems with hybrid hydrogels. (**A**) Fabrication and multiscale bioadhesion of HA–Cat hydrogels. (**B**) Release profile of BSA from both HA–Cat and MeHA hydrogels. (**C**) Sustained protein release from HA–Cat hydrogels controlled by Cat-protein interaction. Reproduced with permission from Zhang et al. [107]. (**D**) Crosslinking mechanism and potential applications of PAADOPA/PAAm hydrogels. (**E**) Cumulative drug release from PAAm and PAADOPA/PAAm hydrogels. (**F**) Viability of HUVECs assessed after treatment with PAAm and PAADOPA/PAAm hydrogels. Reproduced with permission from Xu et al. [289]. (**G**) Synthesis scheme for SIPN and CP hydrogels. (**H**) Hydrogel protein encapsulation efficiency (EE%). (**I**) Lysozyme release profiles from 10MAC-A hydrogels under various pH, temperature, and GSH conditions. Reproduced with permission from Dutta et al. [293]. (**J**) Multicomponent microspheres with DOX-loaded ALG shell and ALR-loaded GelMA cores for treating post-surgical liver cancer and promoting liver regeneration. (**K**) Synthesis process of MCMs. (**L**) Cumulative DOX release from MCMs in PBS at varied pH. (**M**) Cumulative BSA-FITC release from MCMs at different pH levels. Reproduced with permission from Zhong et al. [299].

Hybrid hydrogels hold significant promise in drug delivery. One example is the development of polysaccharide-based hydrogels (HA–Catechol or HA–Cat) mimicking mussel adhesion and enhancing interactions with host tissues (Figure 6A,B) [107]. This hydrogel not only improves adhesion but also facilitates the prolonged release of protein-based biomolecules, such as the osteoinductive BMP-2 protein, promoting stem cell osteogenic differentiation (Figure 6C).

For topical therapy, a nature-inspired PAA-DOPA/PAM hydrogel was designed to prevent adhesion damage under humid conditions (Figure 6D) [289]. This hydrogel exhibited a 3.2 times higher adhesion strength than traditional PAM hydrogels, with excellent biocompatibility and controlled release of encapsulated drug molecules (Figure 6E,F). In a different approach, a sandwich-like system consisting of nanofibers and PDA-SiNFs reinforced hydrogels was created for the sustained, controlled release of therapeutic agents [290]. Superabsorbent polymer compositions (SAPCs) were developed for amoxicillin drug delivery to address peptic and duodenal ulcers induced by Helicobacter pylori.

Smart microgel particles, responsive to temperature and pH changes, were synthesized for controlled insulin delivery [291]. A novel investigation introduced pH-responsive PLGA nanoparticle composite microcapsules designed for the oral delivery of insulin [292]. Findings from in vitro studies indicated that these composite microcapsules maintained their structural integrity in the stomach, underwent gradual dissolution in the small intestine, and eventually almost completely dissolved in the colon. Copolymeric hydrogels, obtained through NIPAAm/CMC copolymerization, were crosslinked for lysozyme delivery, showing varying swelling and release characteristics (Figure 6G–I) [293]. A hybrid hydrogel system based on CMC and carboxymethyl polyvinyl alcohol (CMPVA) grafted copolymer (CMC-g-CMPVA) was developed for drug delivery and tissue engineering, exhibiting biocompatibility and high survival rates for living cells [294]. Stimuli-responsive hydrogels, fabricated from bacterial cellulose (BC)-g-PAA using electron beam irradiation, served as an oral delivery system for proteins without involving toxic crosslinking agents [295].

Microwave-assisted graft copolymerization led to BC-g poly(acrylic acid-co-acrylamide) hydrogels, exhibiting pH-sensitive drug release profiles suitable for potential oral, controlled release drug delivery in the lower gastrointestinal tract [296]. Additionally, copolymeric hydrogels, obtained by the graft copolymerization of stimuli-responsive polymers on various starches, showcased potential as prolonged drug delivery vehicles. For instance, starch-based magnetite nanohydrogels (MNHGs), created by grafting NIPAm and maleic anhydride (MA) monomers onto modified magnetite nanoparticles, enable targeted doxorubicin delivery [297]. These responsive hydrogels, utilizing magnetite nanoparticles, exhibit dual-responsive drug release with an enhanced efficacy at specific pH and temperature conditions.

Hybrid hydrogels have also emerged as a promising platform to enhance existing cancer treatments, with a focus on targeted and localized drug deliveries, taking advantage of the unique characteristics of the tumor microenvironment, such as a low pH and hypoxia. Co-axial hydrogel fibers were developed to facilitate both affinity and controlled diffusion in drug release [298]. The main core consisted of a dopamine-modified Alg hydrogel loaded with chemo-therapeutic model drugs (DOX or gemcitabine) for affinity-controlled release. This approach showed promise both in vitro and in vivo. Zhong et al. conducted a study on multicomponent microspheres (MCMs) designed for post-surgical liver cancer treatment and regeneration (Figure 6J,K) [299]. Utilizing a microfluidic platform, they created MCMs with a sodium alginate (Alg) shell and gelatin methacrylate (GelMA) cores, enabling the dual-drug delivery of doxorubicin and liver regeneration augmenter. The unique “particle-in-particle” structure facilitated rapid doxorubicin release and sustained augmenter release, showcasing its significant efficacy in postsurgical tumor eradication and liver regeneration promotion (Figure 6L,M).

Another innovative hydrogel system, CS-conjugated gallic acid/Fe3^+^ (CSG/Fe3^+^), was designed to prevent high tumor recurrence rates resulting from surgical trauma [300]. The hydrogel, loaded with DOX, demonstrated a synergistic photothermal-chemo tumor-inhibited performance, effectively eliminating remnants of cancer cells recruited due to surgical trauma. The encapsulation of dual anticancer drugs represents a novel approach to enhance the target specificity and efficacy of these hydrogels.

Combining photothermal and multidrug chemotherapy has proven effective in treating cancerous cells or tissues. However, the penetration depth of phototherapy is limited, and many commonly used photo-absorbing agents are non-biodegradable and potentially toxic. Rezk et al. addressed this concern by developing a pH-responsive Alg-PDA polymeric system for selective and localized delivery of BTZ, providing an effective and biocompatible photo-absorbing agent [301]. Additionally, hydrogels composed of poly(glycerol sebacate)-co-PEG-g-catechol and ureido-pyrimidinone-modified gel exhibited an excellent NIR and pH responsiveness. These hydrogels demonstrated in vivo hemostasis of skin trauma and a high efficacy against methicillin-resistant *Staphylococcus aureus* (MRSA).

A novel self-repairing hydrogel adhesive has been formulated to address drug-resistant infections and address skin defects [122]. This injectable hydrogel is composed of poly(glycerol sebacate)-co-poly(ethylene glycol)-g-catechol, crosslinked through catechol–Fe^3+^ coordination, and gelatin modified with ureido-pyrimidinone, crosslinked through quadruple hydrogen bonding responsive to pH and NIR light. Notably, the hydrogel demonstrates outstanding anti-oxidation properties. In vivo tests have showcased its effectiveness in hemostasis, wound closure, and superior healing when compared to both medical glue and surgical sutures. This innovative solution holds great promise for treating skin trauma.

PGS-based systems have been developed for targeted anticancer drug delivery by incorporating fluorouracil (5-FU) into a matrix of PGS-PEG/CS-co-PEG, subsequently coated with iron oxide (PGS PEG/CS-PEG@Fe_3_O_4_) [302]. In these studies, two different rates of 5-FU release were achieved by mixing CS-PEG with PGS-PEG, forming CS-PEG under mild conditions by binding PEG to the hydroxyl group of CS. In simulated physiological fluids, PGS-PEG/CS-PEG@Fe_3_O_4_ displayed controlled release, indicating a retention mechanism model where hydrophobic interactions with the drug were stronger than with pure CS or PGS lacking a hydrophobic group. Cytotoxicity studies on HT29 cell lines confirmed enhanced cytotoxicity compared to pure 5-FU, affirming the drug’s activity post-release. Silva et al. utilized coaxial electrospinning to produce PGS/PCL-aligned nanofibers for cartilage tissue engineering, incorporating Kartogenin (KGN) into the PGS core to create coaxial PGS KGN/PCL nanofibers [303]. These nanofibers facilitated a controlled and sustained KGN release, promoting the chondrogenic differentiation of MSCs. Ye et al. developed a self-healing PGS-PEGMEMA/α-cyclodextrin (αCD) hydrogel as a drug delivery vehicle, exhibiting thixotropic properties and biphasic drug release, offering the potential for controlled drug delivery systems [304].

Metal-organic frameworks (MOFs) have been incorporated into hydrogel systems to create hybrid structures. For instance, Javanbakht et al. developed a bio-nanocomposite hybrid hydrogel bead system containing 5-FU encapsulated porous Zn-based MOFs [305]. This system, coated with CMC, demonstrated sustained drug release and a notable toxicity against HeLa cells, showcasing its potential as an anticancer drug delivery system targeting colon cancer. Another hybrid hydrogel-based system was constructed by Schneible et al., incorporating modified graphene oxide (GO) nanoparticles into a Max8 peptide hydrogel for the controlled release of anticancer drugs [306]. Molecular dynamics (MD) simulations were employed to model the drug-loading mechanism, emphasizing the versatility and potential of hybrid hydrogels in advancing biomedical applications. These diverse approaches collectively underscore the ongoing progress in utilizing hydrogels for innovative and effective cancer therapies.

### 9.2. Hybrid Hydrogels for Tissue Engineering

Hybrid hydrogels offer a unique combination of diverse components to construct scaffolds that closely mimic the ECM. These hydrogels provide an optimal environment for cellular growth and tissue regeneration, making them particularly valuable in addressing challenges associated with bone tissue defects and fractures.

**Figure 7:**
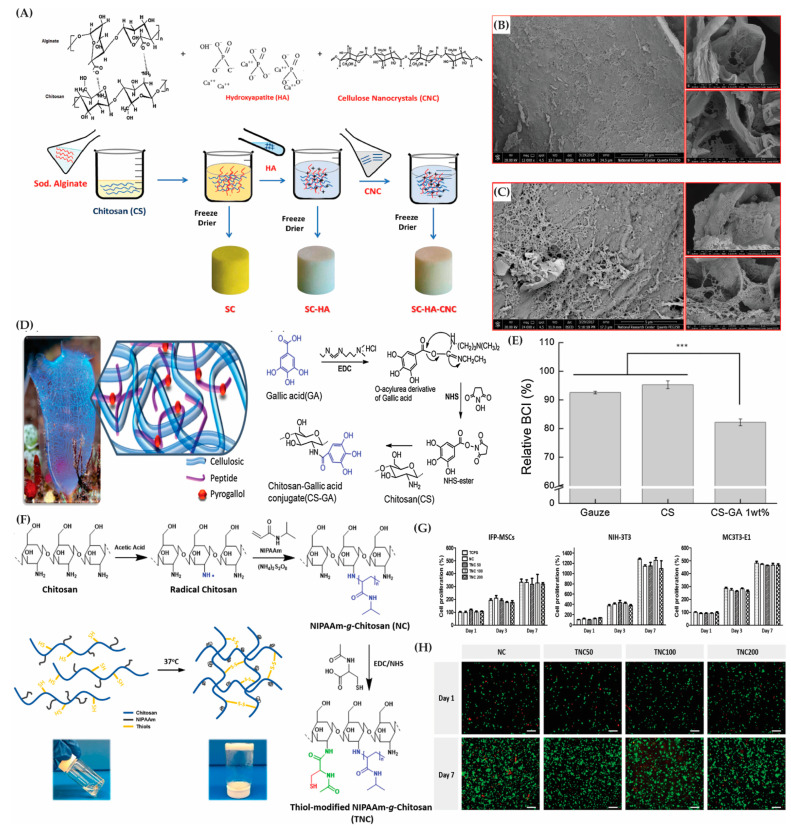
Hybrid hydrogel scaffolds used in tissue engineering. (**A**) Synthesis of chitosan/alginate/hydroxyapatite/nanocrystalline cellulose scaffolds. (**B**,**C**) Scanning electron microscopy (SEM) images of MG63 cells cultured on the scaffolds at 24 h (**B**) and 72 h (**C**) (scale bar: 10 μm). Reproduced with permission from Shaheen et al. [307]. (**D**) Structures of tunicates; tunichrome-inspired chitosan hydrogel (CS-GA) is synthesized via an EDC coupling amidation reaction. (**E**) Enhancement of tensile strength due to the presence of FeCl_3_ and NaIO_4_ crosslinking agents (*** *p* < 0.05, one-way ANOVA). Reproduced with permission from Sanandiya et al. [311]. (**F**) NIPAAm monomer copolymerization with chitosan via free-radical addition and thiol modification employing carbodiimide for NAC and chitosan conjugation. Gelation involves both physical (helix-coil structure) and chemical crosslinking (disulfide bond formation). (**G**) Proliferation of IFP-MSCs, NIH-3T3, and MC3T3-E1 cells in a conditioned medium assessed through MTS assay after 1, 3, and 7 days. Data represented as means ± SD for *n* = 3. (**H**) MC3T3-E1 cell viability within NIPAAm-g-chitosan hydrogels at day 1 and day 7. Live cells exhibit a green fluorescence, while dead cells are stained red. Scale bar: 100 μm. Reproduced with permission from Wu et al. [315].

Various formulations have been explored to create scaffolds conducive to bone tissue engineering. For instance, Shaheen et al. employed chitosan/alginate/hydroxyapatite/cellulose scaffolds synthesized through dicationic crosslinking with calcium chloride (Figure 7A–C) [307]. These scaffolds exhibited improved swelling, mechanical behaviors, and an appropriate porosity compared to existing biomaterials. Additionally, Anuj et al. developed composite hydrogels from PAM, PVA, bioactive glass, and halloysite nanotubes, demonstrating enhanced biomineralization, cell adherence, and cytocompatibility [308]. Gautam et al. employed electrospinning to fabricate a scaffold comprising a tri-polymer (PCL–gelatin–chitosan; PCL–GT–CS) with nano-hydroxyapatite (nHAp) [309]. Their study, utilizing the MTT assay, indicated an enhanced human osteoblast cell viability on the nHAp-modified PCL–GT–CS scaffold compared to the unmodified counterpart. Furthermore, DNA quantification revealed a significantly higher osteoblast proliferation rate on the nHAp-modified scaffold. Meanwhile, Shao et al. utilized a microwave-assisted technology to fabricate a 3D composite scaffold of hydroxyapatite/silk fibroin, demonstrating its excellent mechanical and biological properties [310]. This scaffold promotes optimal cellular responses for effective bone repair.

PGS has emerged as a notable material in bone tissue engineering alongside PHB, PCL, PLA, PLGA, gelatin, CS, and collagen. Despite its soft and elastic nature, typically with an elastic modulus of 0.25 to 1.45 MPa and tensile strength of 0.3 to 1.5 MPa, reinforcing it with ceramic particles or infusing it into a ceramic scaffold effectively bolsters its mechanical properties for bone regeneration [169].

The importance of hydrogels extends also to skin tissue engineering. Nature-inspired hydrogels, with adhesive properties, have shown great potential in facilitating the healing process. A chitosan/gallic acid-based tissue adhesive, inspired by the fibrous structure of tunicates, exhibited twice the adhesion capability, higher platelet adhesion, and blood-clotting capacity (Figure 7D,E) [311]. Balitaan et al. developed injectable, dynamic, and self-healing hydrogels composed of acrylamide-functionalized β-chitin and aldehyde oxidized-alginate, accelerating wound healing in zebrafish significantly [312]. Najafabadi et al. utilized ciprofloxacin (CIP)-loaded gel/PGS fiber membranes for skin tissue engineering [313], assessing different sebacic acid-to-glycerol ratios, and confirming the membrane cytocompatibility with L929 fibroblast cells with potential uses as wound dressings.

Gilarska et al. demonstrated the great potential of injectable hydrogels comprising chemically crosslinked collagen, chitosan, and hyaluronic acid as biomimetic bone scaffolds [314]. The highest concentration (20 mM) of crosslinker resulted in gradual enzymatic degradation and on-site hydrogel formation. The introduction of silica particles into collagen/chitosan hydrogels enhanced the osteogenic differentiation of human bone marrow-derived mesenchymal stem cells, evident in the increased osteogenic factor levels after a 14-day culture compared to pristine hydrogels. Wu et al. employed a disulfide crosslinking strategy to reinforce the mechanical properties of the injectable NIPAAm-g-chitosan hydrogel for tissue engineering (Figure 7F) [315]. Thiol-modified NIPAAm-g-chitosan (NC) hydrogels demonstrated biocompatibility without cytotoxicity in mesenchymal stem cells, fibroblasts, and osteoblasts (Figure 7G,H). The modified thermo-sensitive NC hydrogels exhibited a remarkable nine-fold improvement in their compressive modulus after thiol oxidation into disulfide bonds.

While natural polymers may face challenges in effectively delivering bioactive agents, synthetic scaffolds have proven more successful, offering programmable characteristics like porosity and porous size. Thermosensitive synthetic polymers, including polystyrene, PLLA, PGA, PEG, PCL, and PLGA, can be tailored for specific properties such as gelation temperature, flexibility, release kinetics, and biodegradation features, making them advantageous for designing bone scaffolds. In addressing the challenges of tissue adhesion and mechanical strength, Chen et al. developed an ultra-tough hydrogel with a self-healing ability through dynamic Schiff crosslinking [316]. This study highlighted the superior performance of nature-inspired hydrogels compared to surgical fibrin glue. Additionally, hydrogels composed of modified alginate, PEG-diacrylate, tannic acid, and Pluronic F127 were fabricated for sutureless, adhesive wound-closure patches, outperforming traditional sutures and commercial adhesive pads [317].

The application of hydrogels extends to vascular irregularities, with coated stents playing a crucial role in treating conditions like aneurysms. Obiweluozer et al. developed a non-thrombotic, poly(acrylic acid-co-methyl methacrylate-co-dopamine methacrylamide)/polyurethane (P(AA-co-MMA-co-DMA)/PU) semi-IPN hydrogel-coated stent, emphasizing the benefits of fully wrapped stents in minimizing tissue granulation, suppressing thrombosis, and improving post-operative recovery [318].

Cartilaginous tissue possesses a minimal avascular and aneural chondrogenic density, accompanied by a high water content (around 70%) [319]. This unique tissue plays a crucial role in diarthrosis joints, providing a load-bearing, wear-resistant surface that reduces friction and facilitates effective joint movements. However, its avascularity and low chondrocyte proliferation rate hinder self-repair, making cartilage susceptible to damage from trauma, accidents, or infections, leading to potential cartilage loss. Treating chondral injuries includes therapies like autograft transplantation, periosteal grafts, mosaicplasty, and microfracture. However, clinical studies have not shown consistent, long-term solutions or reliable fibrocartilage production. The complex native tissue structure, characterized by diverse cell morphologies, arrangements, and ECM compositions, poses challenges in producing functional articular cartilage.

The introduction of 3D bioprinting in tissue engineering has made significant strides in replicating articular cartilage anatomy. Researchers assert that this method can create cartilage-like constructs by combining different hydrogels. Li et al. utilized silk and gelatin to fabricate 3D hydrogel scaffolds with varying porosities, incorporating cell seeding for cartilage regeneration [320]. These hydrogels exhibited excellent physicochemical and mechanical properties, along with desirable biodegradation to support cartilage regeneration. Addressing articular cartilage injury, Sun et al. reported a genetic association between growth differentiation factor 5 (GDF5) loci and hip joint dysplasia [321]. They used this genetic insight to create a 3D-bioprinted functional knee cartilage scaffold, demonstrating improved cartilage regeneration and long-term chondroprotective effects. For cartilage tissue regeneration, thermoresponsive bases like PNIPAm or Pluronic, combined with natural polymers such as hyaluronic acid, are commonly used [284].

Injectable adhesive hydrogels, such as (DOPA) amide- or ester-containing poly (2-alkyl-2-oxazoline) (POx)/fibrinogen hydrogels, have been designed to enhance integration at cartilage defect interfaces [322]. These hydrogels demonstrated beneficial long-term lateral cartilage tissue integration, improved degradation, and resulted in robust cartilaginous ECM deposits at the defect site. Inspired by marine creatures, Gan et al. developed a GelMA hydrogel with good toughness, resilience, biocompatibility, and controlled release kinetics [323]. This hydrogel facilitated the in vivo regeneration of cartilage tissue when loaded with chondroitin sulfate or TGF-β3. Additionally, Wang et al. produced a PEG-based composite hydrogel mimicking native articular cartilage with zone-dependent nonlinear and viscoelastic mechanical properties [324].

Enzymatically cross-linkable hydrogels offer potential in tissue engineering and drug delivery. Ziadlou et al. created injectable composite hydrogels with hyaluronic acid–tyramine (HA–Tyr) and regenerated silk-fibroin (SF), demonstrating strong mechanical properties and encouraging chondrogenic matrix production [325]. Ren et al. developed injectable, degradable hydrogels composed of polysaccharides [326]. These hydrogels, incorporating hydroxyapatite (HAp) nanoparticles and calcium carbonate microspheres (CMs) under physiological conditions, enhanced the in vitro synthesis of GAGs and type II collagen. Innovative approaches in tissue engineering address the challenges posed by the avascular and aneural nature of cartilage tissue, utilizing diverse hydrogels, scaffolds, and 3D bioprinting techniques for enhanced regeneration and functional restoration.

A partial liver resection stands as an established treatment for hepatic disorders; nonetheless, challenges persist in the form of surgical bleeding, intra-abdominal adhesion, and rapid liver regeneration, contributing to the associated morbidity and mortality. Addressing these challenges, Li et al. conducted a study introducing a biomimetic hybrid hydrogel comprising oxidized hyaluronic acid, glycol chitosan, and conditioned medium derived from menstrual blood-derived stem cells (MenSCs-CM) [327]. The hydrogel, formed through a reversible Schiff base, exhibited injectability and self-healing capabilities. Notably, it demonstrated hemostasis, anti-infection properties, tissue adhesion, and controlled cargo release. In vivo studies showcased the hybrid hydrogel’s ability to immediately cease acute bleeding in a partial liver resection, reduce intra-abdominal adhesion, and enhance hepatic cell proliferation and tissue regeneration through the controlled release of cytokines from MenSCs-CM. These findings position the biomimetic hybrid hydrogel as a promising candidate for applications in partial liver resection.

For in vitro liver fabrication, a significant challenge is effectively culturing hepatocytes [328]. Alginate-based, 3D-bioprinted scaffolds offer promise by enabling successful hepatocyte culture for up to two weeks while preserving their phenotype [329]. Additionally, 3D-printed scaffolds present new opportunities for creating functional liver tissues. Recent developments include an injectable hydrogel composed of glycyrrhizin, alginate, and calcium, which maintained the proliferation and liver-specific functions of hepatic cell lines [330]. Fibrin hydrogels, in conjunction with PLGA and hepatocytes, formed implantable liver tissues with hierarchical vascular networks [331]. Electrospun nanofibers composed of PCL and chitosan demonstrated the ability to promote the in vitro differentiation of human somatic stem cells into hepatocytes [332]. This highlights the promising potential of PCL/chitosan nanofiber scaffolds for the regeneration of liver tissue. Synthetic material PAA and its derivatives show promise in altering the growth kinetics of adhesion patches at the hepatocyte cell–substrate interface. Conjugating PAA and polyethyleneimine with elastin-like polypeptides influenced the morphology, aggregation, and differentiation function of primary rat hepatocytes [333].

In addressing myocardial infarction (MI), where adult cardiomyocyte regeneration is limited, high morbidity, mortality, and recurrence rates persist. Injecting cells for MI treatment faces challenges such as cellular anchorage and leakage [334]. To address this, a fabricated cardiac patch (ECP) emerges as a potential alternative [335]. Wang et al. developed a mussel-inspired conductive ECP cryogel composed of GelMA, PEGDA, and dopamine-coordinated polypyrrole nanoparticles [336]. In a rat model, this cryogel showed improved fractional shortening, ejection fraction, and a reduced infarct size. Due to its low solution viscosity, PGS has been blended with other polymers for spinnability (for electrospinning). PGS blended with PCL and spun as a fiber mat was utilized in cardiac tissue engineering. Its recent optimization includes electrospinning a patterned PGS/PCL fiber mat on a Teflon-coated silicon wafer with topographical features [337]. In vitro experiments demonstrated the successful alignment of C2C12 myoblasts and neonatal rat cardiomyocytes after 24 h, along with enhanced cell-to-cell communication via connexin43 expression. Rai et al. alternatively functionalized PGS-based fiber mats by chemically conjugating VEGF, facilitating the attachment, growth, and proliferation of various cell types compared to unfunctionalized scaffolds [338]. Shape-memory polymers were explored to create a self-expandable tabular form under NIR irradiation, utilizing a polydopamine nanosphere (PDN)-loaded poly(acrylic acid-co-methyl methacrylic acid) or P(AA-co-MMA)@PDN nanocomposite hydrogel [339]. This material is efficiently heated (reaching 70–80 °C in 6 s) when exposed to NIR. It may be introduced into arteries through minimally invasive surgery, attaching to a catheter temporarily. Further research in animal and other models is crucial to validate its potential. McGann et al. developed three high-molecular-weight RLPs able to form elastic hybrid hydrogels when crosslinked with a PEG-vinyl sulfone crosslinker through a Michael-type addition reaction [174]. The resulting hydrogels were able to encapsulate human aortic adventitial fibroblast cells under mild conditions. Over a 7-day culture period, the encapsulated cells maintained their viability and exhibited a spread morphology resembling a natural fibroblast phenotype. This approach holds potential for cardiovascular tissue engineering materials.

Considering blood vessels’ crucial role, vascular grafts are commonly used in cardiovascular disease cases. A highly stretchable artificial blood vessel is desired for microvascular grafts. A coating of dopamine-grafted heparin to an Alg/PAM double-network hydrogel tube has been applied, enhancing the hemocompatibility without compromising the mechanical performance [340]. This hydrogel tube demonstrated an improved blood endothelial cell attachment and maintained its mechanical strength. Blends of PGS and PCL have also been found suitable for creating vascular grafts. Electrospinning of PGS/PCL in a 1:1 ratio produced small-diameter tubular grafts, with a 2 mm internal diameter and an average fiber outer diameter of 3.94 ± 1.39 and 5.57 ± 1.55 μm) [341]. These grafts exhibited mechanical properties and suture retention similar to native human arteries. Hydrogel patches for cardiovascular diseases require durability, good cardiomyocyte attachment, and synchronous contraction. They can be explored further for clinical applications, potentially being delivered through a catheter with minimal surgical invasion. Hollow scaffolds, fabricated with nature-driven materials and approaches, offer additional avenues for development.

Hybrid hydrogels are also applied in non-targeted tissue engineering. A self-healing poly(N-methylol acrylamide)/PVA hydrogel mimicked muscles at the macroscopic level [342]. Self-healing hydrogels composed of polypeptide and peptide nanofibers exhibited remarkable mechanical properties, thixotropic ability, biocompatibility, and controlled enzymatic degradation [343]. An HA–Catechol coating platform enhanced salivary gland tissue engineering by recapitulating branched structural complexity and heterogeneity in cell populations [344]. The hydrogels incorporate superior toughness and mechanical strength, crucial features for targeting different tissues and reinforcing themselves.

### 9.3. Commercialization and Intellectual Property Protection of Hybrid Hydrogels

Although several hybrid hydrogels have been successfully commercialized and have well-established roles in certain markets, such as contact lenses and wound dressings, the full potential of hydrogels remains untapped. Commercial products for tissue engineering and drug delivery remain limited due in part to high production costs. However, ongoing research and development efforts anticipate further progress.

Patent filings related to hybrid hydrogels have shown considerable growth in the last 5 years. A patent search using either the United States Patent Office (USPTO) Public Search App or Google Patents indicate that a total of 3022 patents disclosing methods for the fabrication of hybrid hydrogels for drug delivery or tissue engineering applications were published between January 2015 and February 2024. Prior to 2018, the number of patents published on these subjects averaged about 106 patents per year, while from 2021 to 2023, the number of patent publications increased to an average of 453 patents per year; representing a four-fold increase in the number of patents filed per year. Universities and research institutes are the top assignees for these patents, but there is also a significant presence of health care venture capitalists and global medical technology companies such as Incept LLC, Covidien Lp, Delsitech Oy, and Edge Therapeutics Inc. Significant examples of recent patent disclosures are summarized below.

Lipke et al. [345] disclosed a PEG-fibrinogen hydrogel for producing tissue and cell cultures from pluripotent and undifferentiated stem cells, offering three-dimensional biomimetic materials with diverse structures such as strings, microspheres, cardiac discs, and macro-tissues. In another disclosure, Shi et al. [346] described a cartilage repair material consisting of a fibrin/hyaluronic acid hydrogel enriched with granulocyte colony-stimulating factor and other pharmaceutical components. Jabbari [347] disclosed methods and compositions of keratin-based hydrogels and injectable formulations that make use of controllable photopolymerizable crosslinking moieties. Kim and Yang [348] described a copolymer hydrogel incorporating crosslinked chitosan, polylactide, fibrinogen, hydrolyzable methacrylate crosslinker, and bioactive agents configured for the sustained release of the one or more absorbed bioactive agents at a predictable release rate. Kim et al. [349] disclosed a temperature-sensitive hydrogel composition comprising chitosan and nucleic acid, notable for its biocompatibility, biostability, and temperature-dependent sol–gel transition. Yi et al. [350] described a method of fabricating macroporous polymeric hydrogel microspheres based on chitosan and polyacrylamide, suitable for carrying biomolecules. Microspheres were fabricated using a micromolding technique that relies on surface tension-induced droplet formation followed by photo-induced polymerization. Osorio et al. [351] disclosed an injectable hydrogel composite formed from chitosan, TEMPO-oxidized cellulose nanofibers, and an acid used for repairing osteochondral defects or meniscus/cartilage lesions or other connective tissue lesions. Yu et al. [352] presented hydrogels comprised of crosslinked hyaluronic acid and silk fibroin, for enhancing the soft tissue and supporting cell proliferation.

## 10. Conclusions and Future Perspectives

Significant progress in the fabrication of hybrid hydrogels for drug delivery and tissue engineering has been made, but numerous challenges still persist. Despite extensive research on hydrogel structures and compositions, a precise understanding of their functions and full potential has not yet been achieved. Commercially available hydrogel products for tissue engineering applications, including drug delivery systems and wound dressings, are currently limited in scope.

Progress in enhancing hydrogel properties for drug delivery has widened the range of drugs and kinetics achievable via hybrid hydrogel systems. However, clinical viability remains a challenge, notably in simplifying their application. For covalently crosslinked hydrogels, releasing crosslinkers within the body could mitigate syringe clogging risks and reduce toxicity, enabling single-syringe mixing. Innovative physicochemical strategies are needed to control gelation and gel–tissue interactions, enhancing injectable hydrogel utility. Challenges persist in expanding the kinetic release profiles, crucial for long-term applications such as insulin delivery. Intelligent hybrid hydrogel systems could regulate drug delivery rates over time, enabling time-varying dose applications. Varied degradation profiles and environmentally responsive segments within hydrogels may address kinetic issues, ensuring efficacy for sensitive molecules like proteins or nucleic acids.

Hydrogels amenable for in situ use hold immense promise as breakthrough materials. Their adaptability to individual patients and multifunctionality position them favorably in the evolving landscape of medical advancements. The inherent biocompatibility and biodegradability of hydrogels, coupled with their capacity to modulate crucial cellular functions like proliferation and differentiation, underscore their attractiveness in tissue regeneration.

The selection of polymers and bioactive substances based on their chemical structure and the impact of this on the desired properties goes beyond simple material combinations to enhance the functionality. The evolution of synthetic polymers has ushered in stimulus-responsive and mechanically robust hydrogels, expanding their applications beyond traditional roles as capsule substrates for cells and drugs. From skin and blood vessels to the robust tissues of bones, hydrogels exhibit versatility in their utility.

The integration of longer-term, disease-like animal models and comprehensive evaluations of the intricate interplay between hydrogel degradation, DDS behaviors, and tissue regeneration processes holds the promise of expediting clinical applications. Hybrid hydrogels, currently employed extensively in targeted cancer chemotherapy and tissue engineering, are poised for further diversification.

Further research on hybrid hydrogels necessitates a focus on drawing inspiration from biological motifs and structures that closely mimic the intended environments. Innovations in facile synthesis methods, particularly one-step processes, will be instrumental in unlocking the full potential of hybrid hydrogels, enabling the preparation of new biomaterials with tunable properties tailored for specific applications and paving the way for their seamless integration into targeted and desired applications in biomedical, chemical, and materials engineering.

## Figures and Tables

**Table 1:** Hybrid hydrogel systems with their applications in drug delivery and tissue engineering.

Hybrid Hydrogels	Biocomponents	Stimuli-Response	Features	Applications	Ref.
SA-PAM	Divalent ions (Zinc)	-	The zinc crosslinked hydrogel exhibits outstanding mechanical strength	Tissue engineering and wound healing	[263]
Chitosan/poly(glutamic acid)/alginate polyelectrolyte complex hydrogels	Piroxicam (PXC)	-	Reduce gastrointestinal side effects of drugs (e.g., piroxicam)	Drug delivery system	[264]
Collagen/alginate/fibrin-based hydrogels	Murine fibroblasts	Temperature	Native soft tissue-like mechanical strength and thermosensitivity	Bone tissue engineering	[265]
PVA/alginate semi-IPN hydrogels	Chondroitin sulfate	-	Enhance chondrogenesis	Cartilage tissue engineering	[266]
Chitosan-PNIPAm hydrogels	hMSCs	Temperature	Sol–gel transition at pH 7.4 and LCST = 32–37 °C	Drug delivery, tissue engineering, and bioadhesive applications	[267]
Alginate-g-P(NIPAM-co-AAPBA)	Insulin	Temperature, glucose	Self-regulating, with the potential for a swift sol–gel transition	Injectable for controlled insulin release	[268]
CPBA-PVA	ATDC5 cells	-	High tensile strain and compressive fracture	Cartilage regeneration	[269]
Hyaluronic acid/collagen/deferoxamine-loaded polydopamine nanoparticles	Deferoxamine (DFO)	-	Desirable mechanical property; enhanced tissue adhesion and injectable properties	Wound healing	[270]
Alginate/dopamine/carboxymethyl chitosan	Fe^3+^	-	Self-healing hydrogel with antimicrobial, adhesive, and conductive properties	Vascular regeneration	[271]
HA/CS/PVA hydrogels	Cefazoline, Theophylline, HaCaT cells	-	85–88% degree of gelation under 15 kGy radiation	Skin tissue engineering	[272]
Collagen–hyaluronic acid	Icariin (Ica)	-	Injectable hydrogel to maintain chondrocyte phenotype	Cartilage repair	[273]
PNIPAm-co-AAC	Chondrocytes, Dexamethasone, TGF β3	Temperature	An injectable hydrogel for promoting chondrogenesis and neocartilage formation	Cartilage tissue engineering	[274]
Alginate–chitosan incorporated with bacterial cellulose	Bovine serum albumin (BSA)	-	Promote osteogenic differentiation	Bone tissue engineering	[275]
Oxidized alginate–gelatin	Chondrocytes	-	Anti-inflammatory property to enhanced 3D printability; promote chondrogenic differentiation	Cartilage tissue regeneration	[276]
Nano-hydroxyapatite/PLGA/Dex	MC3T3-E1 Cells	-	Injectable hydrogel with tissue adhesive properties	Bone tissue engineering	[277]
Epsilon-polylysine-modified cellulose/γ-PGA double network hydrogel	ε-Polylysine (ε-PL)	-	Antibacterial property with excellent biocompatibility	Tissue engineering	[278]
Carbon dots/gelatin/carboxymethyl cellulose	Curcumin, doxorubicin	pH	Superior anticancer effect	Drug delivery system for cancer treatment	[279]
P(NIPAAm-co-PAA-co-MA-PEG-co-HEMA-oTMC)	Cardiosphere-derived cells	Temperature, pH	Sol–gel transition at pH 7.4 and LCST = 37 °C	Cell carriers for cardiac cell therapy, ocular drug delivery	[280]
PNIPAAm-gelatin	Cardiomyocytes, cardiac fibroblasts	Temperature	Sol–gel transition at pH 7.4 and LCST = 55 °C	Cardiac cell delivery and tissue engineering	[281]
Chitosan–ghitin nano-whiskers	Aspirin (ASA)	Temperature	Extracellular matrix imitation ability	Tissue regeneration	[282]
(PNIPAAm-co-IA)-CS	Doxorubicin	Temperature, pH	Sol–gel transition at pH 6.5 and LCST = 37 °C	Anticancer drug delivery	[283]
PNIPAm/HA hydrogels (containing CS-g-AA-coated PLGA or PLGA-ACH microparticles)	Melatonin, MSCs	Temperature	PLGA-ACH microparticles act as carriers of melatonin and reduce PNIPAm/HA syneresis	Drug delivery system and cartilage tissue engineering	[284]
CS/hyperbranched PEG	Adipose-derived MSCs	-	Injectable hydrogel with excellent mechanical feature, rapid gelation, and extended degradation profile	Cartilage tissue engineering	[285]
Sodium alginate/carboxymethyl bacterial cellulose (SA-CMBC)	Fibroblasts	-	Double-network injectable hydrogel with excellent mechanical property	Bone tissue engineering	[286]
Fe_3_O_4_ nanoparticles crosslinked polyethylene glycol hybrid chitosan (mCS-PEG) gel beads	Nanosized Rifampicin (nano-RIF)	pH, magnetic	Hybrid gel beads with dual-responsive assets in nanodrug delivery	Drug delivery (e.g., Rifampicin) application	[287]
Alginate/MXene-based hydrogels	Ag NPs	Photo, magnetic	Excellent antimicrobial feature and precise control release of encapsulated substances	Drug delivery and wound healing	[288]

## Data Availability

Not applicable.
